# A pathogen branched-chain amino acid catabolic pathway subverts host survival by impairing energy metabolism and the mitochondrial UPR

**DOI:** 10.1371/journal.ppat.1008918

**Published:** 2020-09-30

**Authors:** Siraje Arif Mahmud, Mohammed Adnan Qureshi, Madhab Sapkota, Mark W. Pellegrino

**Affiliations:** Department of Biology, University of Texas Arlington, Arlington, Texas, United States of America; university of washington, UNITED STATES

## Abstract

The mitochondrial unfolded protein response (UPR^mt^) is a stress-activated pathway promoting mitochondrial recovery and defense against infection. In *C*. *elegans*, the UPR^mt^ is activated during infection with the pathogen *Pseudomonas aeruginosa*—but only transiently. As this may reflect a pathogenic strategy to target a pathway required for host survival, we conducted a *P*. *aeruginosa* genetic screen to uncover mechanisms associated with this temporary activation. Here, we find that loss of the *P*. *aeruginosa* acyl-CoA dehydrogenase FadE2 prolongs UPR^mt^ activity and extends host survival. FadE2 shows substrate preferences for the coenzyme A intermediates produced during the breakdown of the branched-chain amino acids valine and leucine. Our data suggests that during infection, FadE2 restricts the supply of these catabolites to the host hindering host energy metabolism in addition to the UPR^mt^. Thus, a metabolic pathway in *P*. *aeruginosa* contributes to pathogenesis during infection through manipulation of host energy status and mitochondrial stress signaling potential.

## Author summary

The host engages multiple defense mechanisms in order to survive pathogen infection. Some pathogens have devised strategies to counteract these defense mechanisms to promote their success during infection. The mitochondrial unfolded protein response (UPR^mt^) is classically involved in resolving mitochondrial dysfunction but is also necessary to protect the host during infection with bacterial pathogens such as *Pseudomonas aeruginosa*. In *C*. *elegans*, while *P*. *aeruginosa* activates the UPR^mt^, chronic infection dampens this stress response pathway. Employing a genetic screen, we find that the novel *P*. *aeruginosa* acyl-CoA dehydrogenase FadE2, involved in the catabolism of the branched-chain amino acids valine and leucine, is associated with the repression of the UPR^mt^ that limits host survival. Our findings suggest that competition for nutrients between the host and pathogen may dictate UPR^mt^ activity and ultimately, host survival during infection.

## Introduction

Mitochondria supply cellular energy in the form of ATP through the actions of the tricarboxylic acid (TCA) cycle and oxidative phosphorylation (OXPHOS). OXPHOS is performed by the electron transport chain residing in the mitochondrial inner membrane. Mitochondria face various challenges including a proteome that is encoded by two genomes requiring coordinated gene expression and assembly, as well as free radical damage that can disrupt mitochondrial proteostasis, and the accumulation of toxins.

Cells use a variety of mechanisms to mitigate mitochondrial stress including the mitochondrial unfolded protein response (UPR^mt^) [1–3]. The UPR^mt^ is activated during stress in order to help restore mitochondrial homeostasis through the transcriptional regulation of a variety of protective genes. In *C*. *elegans*, the bZIP transcription factor ATFS-1 mediates the UPR^mt^ and is regulated by mitochondrial import efficiency [4, 5]. ATFS-1 contains a mitochondrial targeting sequence and is imported into healthy mitochondria where it is turned over via protease-mediated degradation. Import efficiency is reduced in dysfunctional mitochondria hindering the entry of ATFS-1 into the organelle. As a result, ATFS-1 accumulates cytoplasmically during mitochondrial stress and, because it also has a nuclear localization sequence, is imported into the nucleus to transcriptionally regulate a diverse set of genes which promote mitochondrial recovery [5].

Mitochondria mediate various defenses against pathogen infection including the regulation of innate immunity [6]. The UPR^mt^ orchestrates its own innate immune response during infection with pathogens that target mitochondrial function [7–9]. Consistent with a role in regulating innate immunity, the UPR^mt^ is both required and sufficient to protect the host during infection [7, 9, 10]. Among the bacterial pathogens that can activate the UPR^mt^ is the opportunistic pathogen *Pseudomonas aeruginosa* [7]. *P*. *aeruginosa* has long been used as a model in the study of host-pathogen interactions using *C*. *elegans* as a host [11]. *P*. *aeruginosa* has multiple means of subverting the health of its *C*. *elegans* host including deterioration of gut epithelial integrity resulting from pathogen colonization (slow killing; [12]), lethality from pathogen-derived hydrogen cyanide (fast killing; [13]), and death by hypoxia via production of iron-sequestering siderophores (liquid killing; [14]). While *P*. *aeruginosa* gut colonization activates the UPR^mt^ in *C*. *elegans*, recent evidence shows that chronic *P*. *aeruginosa* infection can subsequently repress this protective pathway [15]. The repression occurs at least in part through manipulation of the *C*. *elegans* bZIP transcription factor ZIP-3 which functions as a negative regulator of the UPR^mt^ [15]. How *P*. *aeruginosa* executes this repression of the UPR^mt^ is currently not known.

We conducted a genetic screen of *P*. *aeruginosa* mutants to uncover microbial effectors that have a role in modifying the UPR^mt^. Using this approach, we found that a loss of function mutation in the *P*. *aeruginosa* gene *fadE2* leads to enhanced UPR^mt^ activity throughout infection resulting in extended host survival. FadE2 encodes an acyl-CoA dehydrogenase with a preference for isobutyryl-CoA and isovaleryl-CoA, metabolic intermediates produced during the breakdown of the branched-chain amino acids (BCAA) valine and leucine, respectively. Interestingly, host energy metabolic pathways are reduced during infection with *P*. *aeruginosa* due to the presence of FadE2. Consequently, loss of FadE2 restores host energy metabolism and the ability of the host to activate the UPR^mt^. Our results suggest that *P*. *aeruginosa* FadE2 restricts valine and leucine catabolites during infection which antagonizes host metabolism, UPR^mt^ activity and host survival. Consistently, supplementation with valine or leucine is sufficient to counteract the repression of the UPR^mt^ and extend host survival during *P*. *aeruginosa* infection. Thus, we propose that by limiting valine and leucine catabolites, *P*. *aeruginosa* FadE2 antagonizes host survival by altering host metabolism and the activity of the UPR^mt^.

## Results

### A genetic screen to identify *P*. *aeruginosa* effectors that repress UPR^mt^ signaling

**Fig 1:**
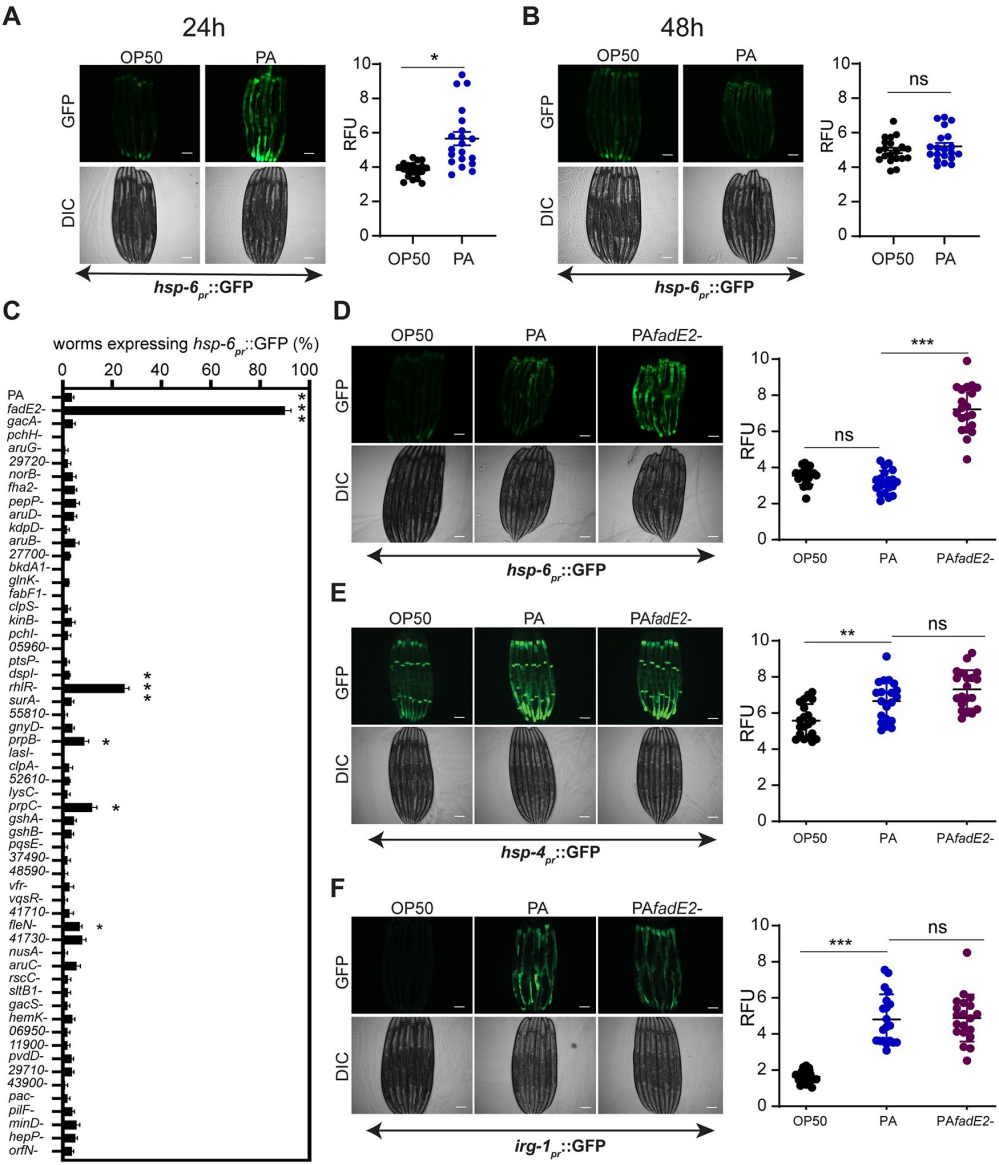
*P*. *aeruginosa* FadE2 mediates the repression of the UPR^mt^. (A, B) *hsp-6*_*pr*_::GFP animals exposed to *E*. *coli* OP50 or wild-type *P*. *aeruginosa* (PA) for (A) 24 hrs or (B) 48 hrs with quantifications of fluorescence. RFU: Relative Fluorescence Units. (C) Quantification of proportion of animals expressing *hsp-6*_*pr*_::GFP animals exposed to various *P*. *aeruginosa* transposon insertion mutants following 48 hrs of infection. (D-F) *hsp-6*_*pr*_::GFP (D), *hsp-4*_*pr*_::GFP (E), and *irg-1*_*pr*_::GFP (F) animals grown in the presence of *E*. *coli* OP50, wild-type *P*. *aeruginosa* (PA), or *fadE2-* for 48 hrs with quantifications of fluorescence. RFU: Relative Fluorescence Units. (A-F) Shown is the mean ± SEM (n≥20 worms). Scale bar is 100 μm for all images. *** denotes p<0.001, ** denotes p<0.01, *denotes p<0.05 using Student’s *t*-test.

In *C*. *elegans*, the activity of the UPR^mt^ can be examined using the transgenic strain SJ4100 *hsp-6*_*pr*_::GFP [16]. The expression of the mitochondrial chaperone *hsp-6* is increased during mitochondrial stress as part of the UPR^mt^ [5]. As expected, exposure of SJ4100 animals to the wild-type PA14 strain of *P*. *aeruginosa* resulted in a modest activation of the *hsp-6*_*pr*_::GFP reporter after 24 hrs, consistent with an activation of the UPR^mt^ (Fig 1A) [7]. However, the level of *hsp-6*_*pr*_::GFP activation decreased with continued exposure to *P*. *aeruginosa* until the signal was completely absent 48 hrs post-infection (Fig 1B). We screened a *P*. *aeruginosa* transposon insertion mutant library [17] in an effort to identify *P*. *aeruginosa* effectors responsible for the repression of the UPR^mt^ during infection. We focused on *P*. *aeruginosa* mutants that were previously identified as potential virulence factors [17]. Using this screening approach, we discovered that a mutation in the *P*. *aeruginosa* gene PA14_31580 resulted in sustained expression of *hsp-6*_*pr*_::GFP at 48 hrs of infection (Fig 1C and 1D). Furthermore, SJ4100 animals exposed to the *P*. *aeruginosa* PA14_31580 mutant displayed a higher level of *hsp-6*_*pr*_::GFP expression following the initial 24 hrs of infection in contrast to those animals that were exposed to wild-type *P*. *aeruginosa* (S1 Fig). Other *P*. *aeruginosa* mutants also prolonged UPR^mt^ activity during infection, albeit at a lower penetrance than PA14_31580. These include mutations in the quorum sensing receptor *rhlR*, the flagella regulator *fleN*, and the genes encoding 2-methylisocitrate lyase and methylcitrate synthase, *prpB* and *prpC*, respectively. We focused on PA14_31580 considering its highly penetrant effect on the activation of the UPR^mt^. We have since named PA14_31580 as FadE2 based on protein homology and refer to the mutant as *fadE2-* henceforth.

*C*. *elegans* has an innate ability to avoid harmful bacteria as a means of protecting itself in a microbe-rich environment [18]. Potentially, the increases in UPR^mt^ activity that is observed during exposure to *fadE2-* could be due to disruptions in pathogen avoidance leading to enhanced bacterial exposure. However, we observed no difference in pathogen avoidance during infection with wild-type *P*. *aeruginosa* and *fadE2-* (S2 Fig). Therefore, the enhanced UPR^mt^ observed during infection with *fadE2-* is not likely due to differences in pathogen exposure.

Next, we examined whether the effect on the UPR^mt^ during infection with *fadE2-* was specific since other cellular stress responses are also activated during infection with this pathogen. The endoplasmic reticulum (ER) exhibits organellar stress during *P*. *aeruginosa* infection due to the increased production of host secreted anti-microbial agents that results in activation of the ER unfolded protein response (UPR^ER^) [19]. Also, *P*. *aeruginosa* produces the exotoxin ToxA that attenuates host protein translation by ribosylating EF-2 causing activation of a surveillance program mediated by the bZIP transcription factor ZIP-2 [20–22]. We therefore monitored the activities of the UPR^ER^ and ZIP-2 translation surveillance program using transgenic animals expressing transcriptional reporters *hsp-4*_*pr*_::GFP and *irg-1*_*pr*_::GFP, respectively. We observed no further increase in activities of these stress responses (Fig 1E and 1F). Thus, loss of FadE2 function appears to specifically enhance the activity of the UPR^mt^ stress response pathway.

### Mitochondrial activity is impaired during infection with *P*. *aeruginosa* through the actions of FadE2

**Fig 2:**
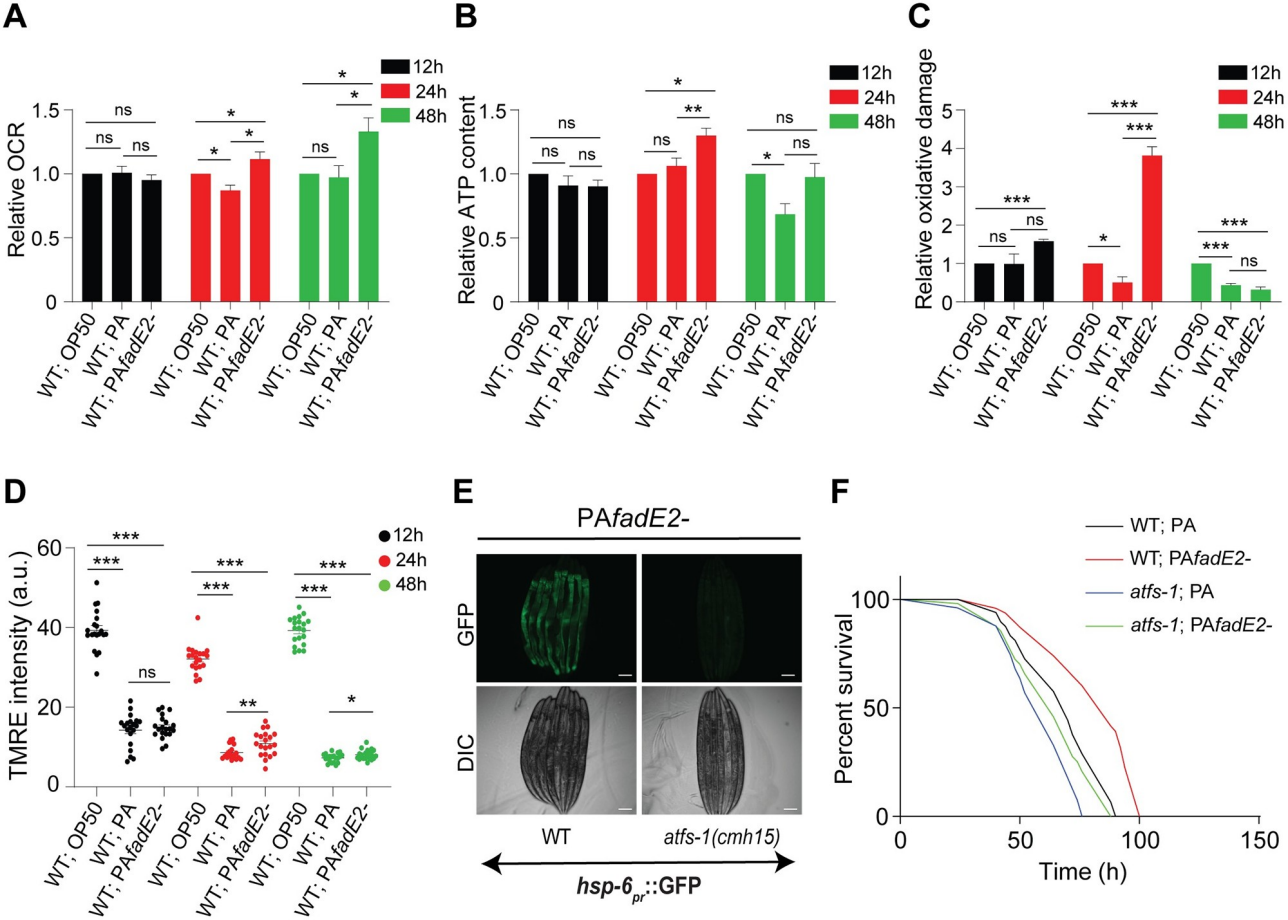
*P*. *aeruginosa* impairs host mitochondrial activity via FadE2. (A-C) Quantification of (A) oxygen consumption rate (OCR), (B) ATP production, (C) level of protein carbonylation for wild-type animals exposed to *E*. *coli* OP50, wild-type *P*. *aeruginosa* (PA) or *fadE2-* for the indicated amounts of time. Shown is the mean ± SEM normalized to total protein content (n = 4 for A and B, n = 3 for C). (D) Quantification of mitochondrial membrane potential using TMRE for wild-type animals exposed to *E*. *coli* OP50, wild-type *P*. *aeruginosa* (PA) or *fadE2-* for the indicated amounts of time. A.U. arbitrary units. (n≥20). (A-D) *** denotes p<0.001, ** denotes p<0.01, *denotes p<0.05 using Student’s *t*-test. (E) *hsp-6*_*pr*_::GFP expression in wild-type and *atfs-1(cmh15)* animals infected with *fadE2-* for 48 hrs. Scale bar is 100 μm. (F) Survival analysis of wild-type and *atfs-1(cmh15)* animals during infection with wild-type *P*. *aeruginosa* (PA) or *fadE2-*. See S2 Table for all survival data statistics.

Exposure to *fadE2-* led to increased UPR^mt^ activity, prompting us to explore mitochondrial function in these animals. We first examined mitochondrial OXPHOS efficiency by monitoring oxygen consumption in wild-type *C*. *elegans* exposed to non-pathogenic *E*. *coli* OP50, wild-type *P*. *aeruginosa* or *fadE2-*. While no differences in oxygen consumption rates (OCR) were observed during the first 12 hrs of exposure, animals infected with wild-type *P*. *aeruginosa* respired less than animals exposed to *E*. *coli* OP50 at 24 hrs of infection (Fig 2A). In contrast, OCR was increased in animals infected with *fadE2-* both at 24 and 48 hrs infection relative to wild-type *P*. *aeruginosa* and even *E*. *coli* OP50 (Fig 2A). Consistent with our OCR findings, ATP production was reduced at 48 hrs of infection with wild-type *P*. *aeruginosa* relative to animals exposed to *E*. *coli* OP50 (Fig 2B). And, infection with *fadE2-* led to greater ATP production relative to animals fed *E*. *coli* OP50 at 24 hrs of infection but returned to wild-type levels at 48 hrs infection (Fig 2B).

Buildup of reactive oxygen species (ROS) can occur with increased mitochondrial activity as a byproduct of oxidative phosphorylation which can alter protein integrity through carbonylation modification. We used the Oxyblot system to assess oxidative damage, an assay which detects carbonylated proteins that can result from ROS accumulation. Interestingly, an increase in oxidative damage was observed at 12 and 24 hrs during infection with *fadE2-*, followed by a sharp decline at 48 hrs post-infection (Fig 2C). Transient increase in ROS levels have been previously attributed to enhanced antioxidant defenses [23]. Consistently, we observed increased levels of the antioxidant glutathione and its derivatives following 48 hrs of infection with *fadE2-* using mass spectrometry (S3 Fig and S1 Table).

We next assayed mitochondrial membrane potential as a reflection of mitochondrial health in our infected animals. As expected, mitochondrial membrane potential was decreased during infection with wild-type *P*. *aeruginosa* relative to animals fed *E*. *coli* OP50 (Fig 2D). However, while mitochondrial membrane potential was also reduced in animals infected with *fadE2-* it nonetheless remained slightly higher than those animals infected with wild-type *P*. *aeruginosa* suggesting a modest improvement in mitochondrial function.

We wondered whether the restoration of the UPR^mt^ during infection with *P*. *aeruginosa fadE2-* promoted host survival in an ATFS-1-dependent manner. As expected, loss of ATFS-1 suppressed the activation of the UPR^mt^ during infection with *fadE2-* (Fig 2E). We next compared the survival of wild-type *C*. *elegans* during infection with wild-type *P*. *aeruginosa* or *fadE2-*. Consistent with previous findings [17], host survival was increased during infection with *fadE2-* relative to wild-type (Fig 2F; *p*-value <0.0001; see S2 Table for statistics pertaining to all survival analyses in this study). And, supporting a role of the UPR^mt^ in mediating this enhanced survivability, loss of ATFS-1 rendered animals hypersensitive to infection (Fig 2F; *p*-value 0.0004).

Importantly, we wished to exclude the possibility that the observed increase in host survival was either due to reduced bacterial viability and/or pathogen virulence. However, we found no differences in the rate of growth or final cell densities for *fadE2-* grown in standard Lysogeny Broth or NGM (S4A and S4B Fig). We then compared various properties associated with pathogen virulence between wild-type *P*. *aeruginosa* and *fadE2-* by first examining biofilm formation but found no significant difference (S4C Fig). Next, we examined two motility behaviors related to bacterial virulence: swarming and twitching. Bacterial motility is a type of migration driven by flagella whereas bacterial twitching is mediated by pili, but neither were significantly affected with loss of FadE2 (S4D and S4E Fig). We also tested for possible differences in lipopolysaccharide (LPS) levels in the absence of FadE2 but found no significant changes (S4F Fig). Lastly, we observed no significant difference in the production of a subset of virulence factors including proteases, elastase, rhamnolipids, cyanide, and pyocyanin (S4G–S4K Fig). Together, our data suggest that the increase in host survival during exposure with *fadE2* is not a result of reduced production of virulence-associated factors or behaviors.

We next performed a rescue experiment by complementing *fadE2-* on a plasmid containing its own promoter to ensure that the effects of *fadE2-* on UPR^mt^ activity and host survival were not due to a secondary mutation. Transient UPR^mt^ activity was restored when animals were infected with the complemented strain (S5A Fig). As well, the susceptibility of *C*. *elegans* to the complemented strain was comparable to that observed when exposed to wild-type *P*. *aeruginosa* (S5B Fig; *p*-value 0.227). Therefore, the effects on the UPR^mt^ and host survival are directly due to the loss of *P*. *aeruginosa* FadE2.

Taken together, our results indicate that *P*. *aeruginosa* FadE2 mediates the suppression of mitochondrial activity and the UPR^mt^ that reduces host survival during infection.

### *P*. *aeruginosa* FadE2 is an acyl-CoA dehydrogenase involved in valine and leucine catabolism

**Fig 3:**
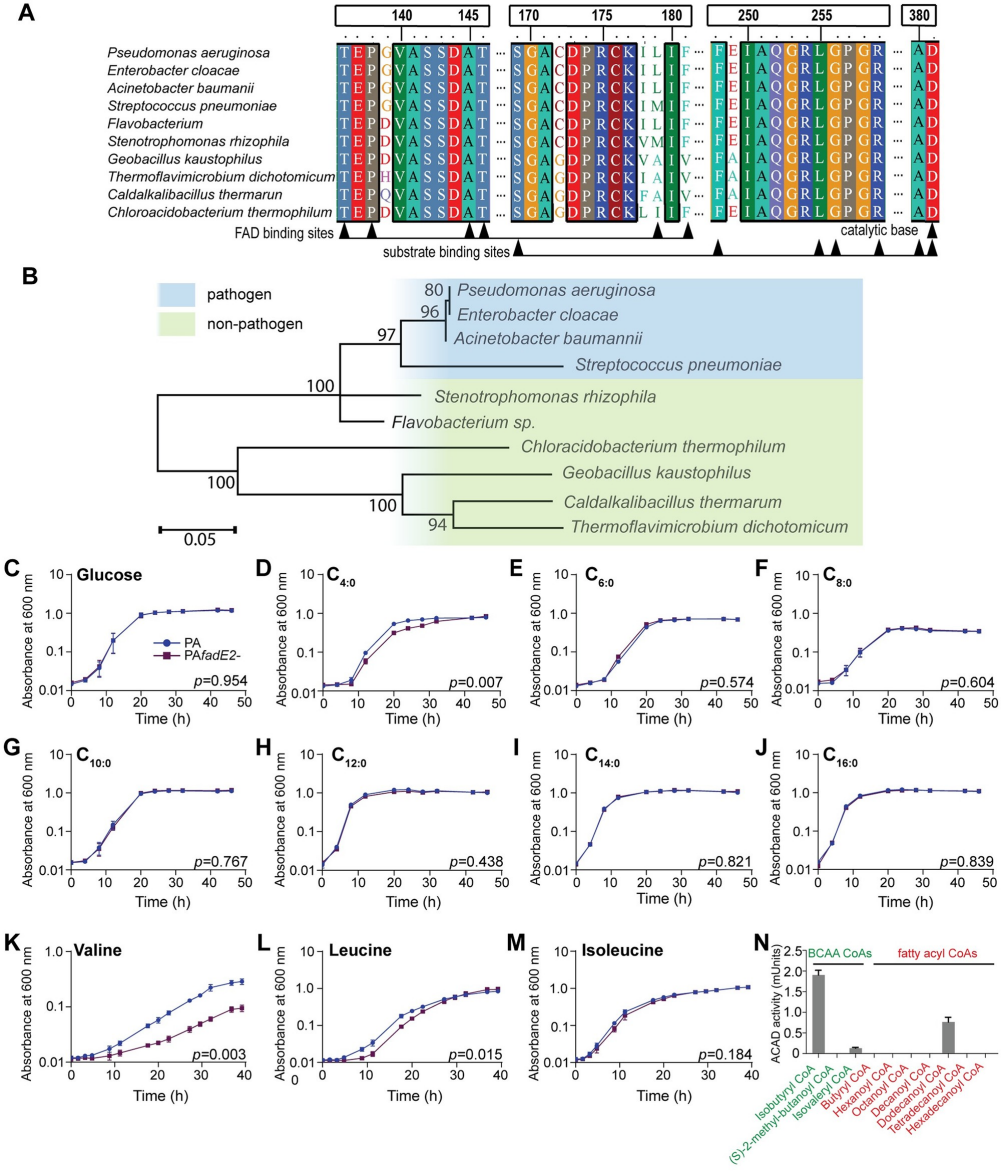
*P*. *aeruginosa* FadE2 is an acyl-CoA dehydrogenase involved in valine and leucine catabolism. (A) Protein alignment of FadE2 with other conserved acyl-CoA dehydrogenase homologs. (B) Phylogenetic analysis of FadE2. Scale bars represents 0.05 substitutions per amino acid position. (C-M) Bacterial growth analysis of wild-type *P*. *aeruginosa* (PA) and *fadE2-* using glucose or various fatty acids or BCAAs as the sole carbon source. Shown is the mean ± SEM (n = 3). *p* values obtained using Student’s *t*-test. (N) Enzyme activity of FadE2 using the indicated CoA esters as substrates. FadE2 activity was measured as described in “Materials and Methods”. Shown is the mean ± SEM of acyl-CoA dehydrogenase activity (n = 3).

The *fadE2* gene encodes a predicted acyl-CoA dehydrogenase, a class of enzyme involved in the breakdown of fatty acids via β-oxidation and also amino acid catabolism (S6 Fig). Acyl-CoA dehydrogenases that are associated with fatty acid breakdown belong to the short-, medium-, long-, and very long-chain acyl-CoA dehydrogenases whereas those that mediate the breakdown of amino acids show preference for the BCAAs valine (isobutyryl-CoA dehydrogenase), leucine (isovaleryl-CoA dehydrogenase) and isoleucine ((S)-2-methyl-butanoyl-CoA dehydrogenase). FadE2 is conserved amongst other uncharacterized acyl-CoA dehydrogenases that are present in a range of bacterial species (Fig 3A). Characteristic features of acyl-CoA dehydrogenases are present in FadE2 including a conserved aspartate active site and binding sites for the flavin adenine dinucleotide (FAD) cofactor. Interestingly, the acyl-CoA dehydrogenases that showed highest homology are present in other human pathogens including *Streptococcus pneumoniae* and *Acinetobacter baumannii* (Fig 3B).

To evaluate whether FadE2 was involved in β-oxidation, we first monitored growth of *fadE2-* in the presence of various fatty acids as the sole carbon source. Apart from observing a mild but significant decrease in growth rate when *fadE2-* was grown in the presence of the short chain fatty acid butyric acid (Fig 3D), no difference in *fadE2-* growth was detected for the remaining fatty acids tested (Fig 3E–3J). We next tested the ability of *fadE2-* to grow in the presence of BCAAs as their sole carbon source. Interestingly, growth of *fadE2-* was dramatically impaired when valine, and to a lesser extent leucine, was used as a sole carbon source (Fig 3K and 3L). No significant difference in growth was detected in the presence of isoleucine (Fig 3M). These results suggest that FadE2 shows a preference for BCAA substrate intermediates of valine and leucine catabolism.

We next recombinantly expressed FadE2 to biochemically validate its substrate specificities using various CoA esters (Fig 3N). Consistent with our growth assays using minimal media, FadE2 displayed a predominant specificity for isobutyryl-CoA, the metabolic intermediate of valine catabolism. Activity was also detected, although to a lesser extent, for isovaleryl-CoA which is the catabolic intermediate of leucine. Interestingly, FadE2 displayed modest activity for dodecanoyl-CoA (Fig 3N), despite no observed difference in *fadE2-* growth when its precursor fatty acid was used as a sole carbon source (Fig 3H).

### *P*. *aeruginosa* FadE2 represses host energy pathways

**Fig 4:**
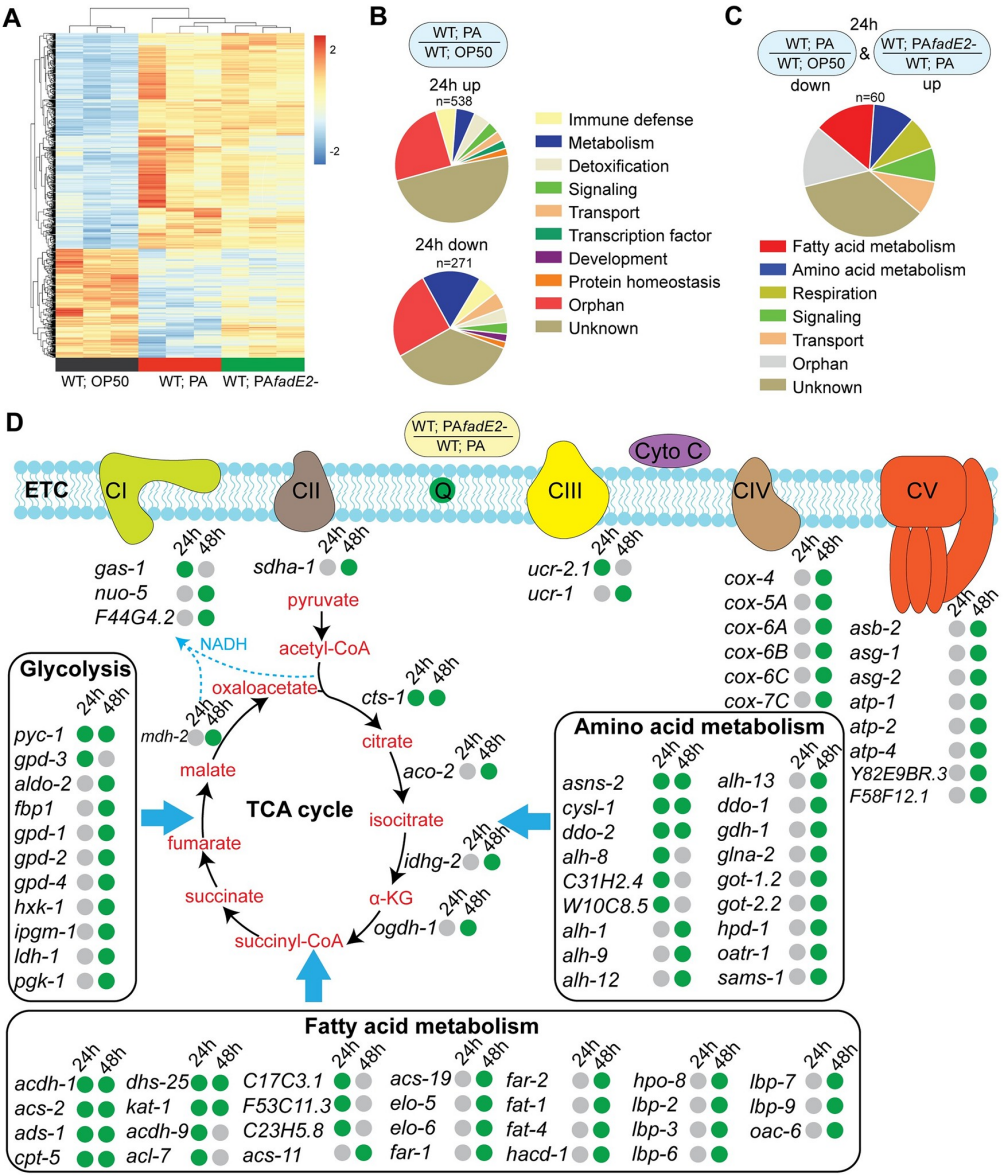
Host metabolic gene expression is repressed with *P*. *aeruginosa* in a FadE2 dependent manner. (A) Heat map representing the change in gene expression patterns of wild-type worms exposed to *E*. *coli* OP50, wild-type *P*. *aeruginosa* (PA) or *fadE2-* for 24 hrs (significantly different at *P*_*adj*_<0.05). (B) Pie chart analysis illustrating the gene categories upregulated and downregulated in wild-type animals infected with wild-type *P*. *aeruginosa* (PA) for 24 hrs relative to those exposed to *E*. *coli* OP50. (C) Pie chart analysis illustrating the gene categories that were downregulated in animals exposed to wild-type *P*. *aeruginosa* (PA) relative to *E*. *coli* OP50 and were upregulated in animals exposed to *fadE2-*. (D) Metabolic network of genes differentially expressed during infection with wild-type *P*. *aeruginosa* (PA) or *fadE2-* for 24 and 48 hrs (n = 3). Green and grey dots represent increase or no change in gene expression, respectively.

We next explored global changes in transcription occurring in the host during infection with *P*. *aeruginosa* in the presence or absence of FadE2 by first comparing *C*. *elegans* genes that were differentially expressed at 24 hrs of exposure to non-pathogenic *E*. *coli* OP50, wild-type *P*. *aeruginosa* or *fadE2-* using RNAseq (Fig 4A and S3 Table). As expected, the expression of genes of various functional categories were both increased and decreased after 24 hrs of *P*. *aeruginosa* infection compared to *E*. *coli* OP50 fed animals (Fig 4B and S3 Table). Interestingly, infection with *fadE2-* increased the expression of many genes that were downregulated during wild-type *P*. *aeruginosa* infection (Fig 4C). In contrast, loss of FadE2 had comparatively less effect on the expression of genes that were upregulated during wild-type *P*. *aeruginosa* infection (Fig 4A). Remarkably, a large proportion of genes whose expression was higher during infection with *fadE2-* were associated with metabolic roles including fatty acid metabolism, amino acid metabolism and respiration (Fig 4C). A similar trend of higher metabolic gene expression during infection with *fadE2-* compared to wild-type *P*. *aeruginosa* was observed at 48 hrs, albeit with a greater number of differentially expressed genes (Fig 4D and S3 Table). We find that multiple genes encoding energy promoting pathways such as glycolysis, amino acid metabolism, and β-oxidation were higher during infection with *fadE2-* relative to those infected with wild-type *P*. *aeruginosa* (Fig 4D and S3 Table). Furthermore, various genes with roles in the tricarboxylic acid (TCA) cycle and mitochondrial OXPHOS were also expressed at a higher level when infected with *fadE2-*. Among the TCA cycle-related genes, we identified *cts-1* (citrate synthase), *idhg-2* (isocitrate dehydrogenase subunit) and *mdh-2* (malate dehydrogenase). Notable OXPHOS genes included *cox-6C/7C* (cytochrome oxidase assembly protein), *cox-4/5A* (cytochrome c oxidase subunit), *sdha-1* (succinate dehydrogenase complex subunit), *coq-1* (Coenzyme Q), *nuo-5* (NADH ubiquinone oxidoreductase), and various genes encoding components of the ATP synthase complex (*atp-1*, *atp-2*, *atp-4*, *asb-2*, *asg-1*, *asg-2*, *F58F12*.*1*).

We wondered whether the increase in metabolic gene expression during infection with *fadE2-* was due to the UPR^mt^. We therefore compared metabolic genes that were differentially expressed during *fadE2-* infection with known ATFS-1-dependent targets [5]. We find that only 7 out of 134 metabolic genes are known to be regulated by ATFS-1, suggesting that their expression is largely independent of the UPR^mt^.

**Fig 5:**
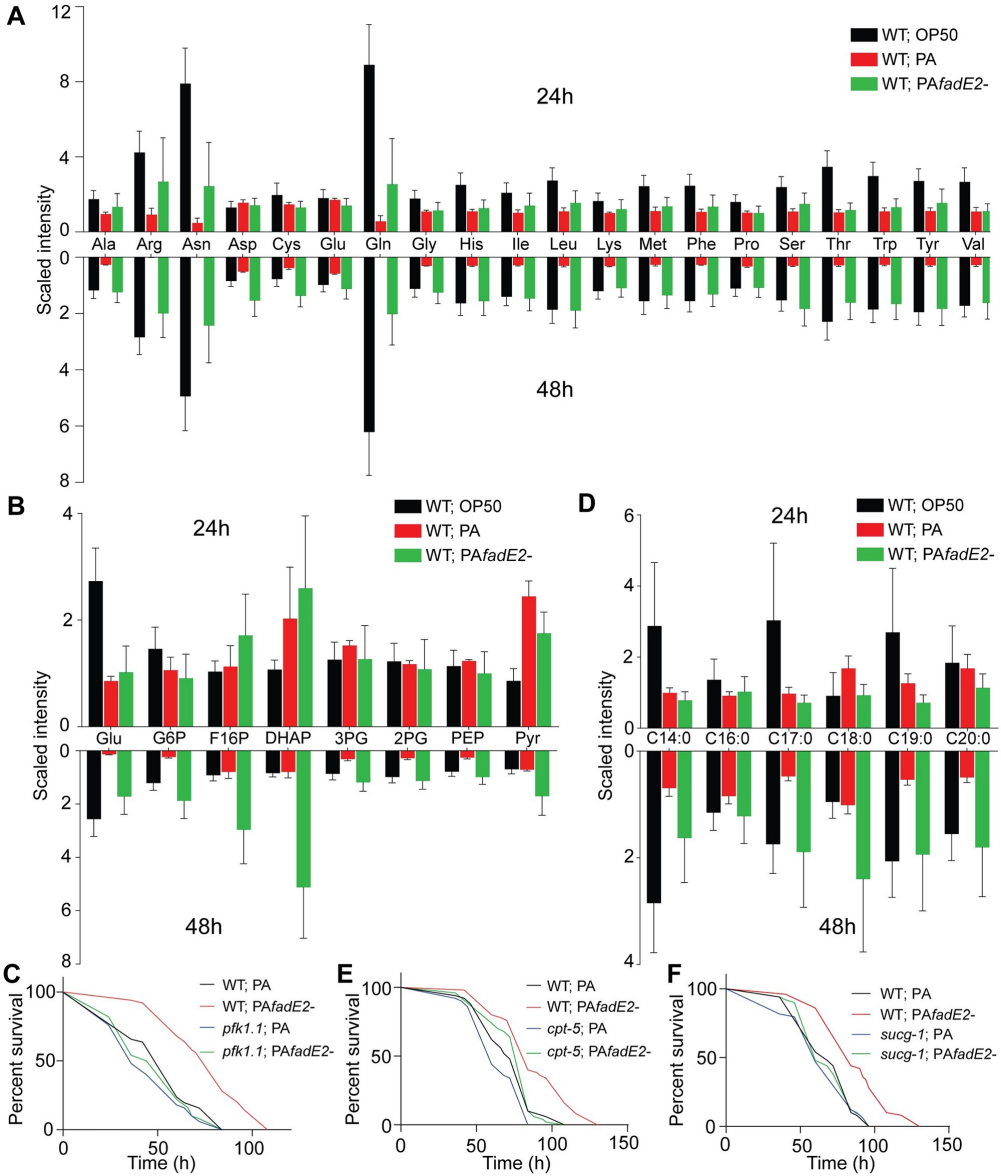
*P*. *aeruginosa* FadE2 mediates the suppression of host energy pathways during infection. (A, B) Quantification of (A) individual amino acids and (B) glycolysis/gluconeogenesis metabolites from wild-type animals exposed to *E*. *coli* OP50, wild-type *P*. *aeruginosa* (PA) or *fadE2-* following 24 and 48 hrs. (n≥4). See S1 Table for statistics. (C) Survival of wild-type or *pfk-1*.*1(ola72)* animals infected with wild-type *P*. *aeruginosa* (PA) or *fadE2-*. (D) Quantification of long-chain and very-long chain saturated fatty acids from wild-type animals exposed to *E*. *coli* OP50, wild-type *P*. *aeruginosa* (PA) or *fadE2-* following 24 and 48 hrs (n≥4). See S1 Table for statistics. (E) Survival of *cpt-5(gk5128)* animals infected with wild-type *P*. *aeruginosa* (PA) or *fadE2-*. (F) Survival of *sucg-1(osa2)* animals infected with wild-type *P*. *aeruginosa* (PA) or *fadE2-*.

Our transcriptomic analysis indicated that expression of genes related to host energy pathways were repressed during infection with *P*. *aeruginosa* in a FadE2-dependent manner suggesting altered host metabolism. We therefore performed a metabolomic analysis of wild-type *C*. *elegans* that were exposed to non-pathogenic *E*. *coli* OP50, wild-type *P*. *aeruginosa* or *fadE2-* for 24 and 48 hrs using non-targeted quantitative mass spectrometry. We observed less overall metabolic difference following 24 hrs of infection (S1 Table) in comparison to the dramatic changes observed following 48 hrs of infection with *fadE2-* (S1 Table). Strikingly, multiple energy-producing pathways were reduced at 48 hrs of *P*. *aeruginosa* infection compared to those fed *E*. *coli* OP50. First, a decrease in abundance of all amino acids was detected during *P*. *aeruginosa* infection (Fig 5A). Interestingly, loss of FadE2 increased levels of all amino acids or even resulted in a restoration back to levels observed when fed *E*. *coli* OP50 at 48 hrs post-infection (Fig 5A). The decrease in total amino acid content during *P*. *aeruginosa* infection may be due to reduced amino acid transport, which is thought to involve the addition of a gamma-glutamyl group by gamma-glutamyl transferase [24]. Indeed, we observed lower levels of gamma-glutamyl amino acids during *P*. *aeruginosa* infection which was increased with *fadE2-* (S7A Fig).

Second, a decrease in metabolites involved in carbohydrate metabolism was also observed in animals infected with *P*. *aeruginosa*. Specifically, glucose and metabolic intermediates of glycolysis/gluconeogenesis were downregulated during infection with *P*. *aeruginosa* and increased with *fadE2-* (Fig 5B). We hypothesized that restored glycolysis might contribute to the extension in host survival observed during *fadE2-* infection. To examine whether glycolysis mediated the extended survival of animals infected with *fadE2-* we used a reduction of function mutant in *pfk-1*.*1* encoding the *C*. *elegans* homolog of the glycolysis rate-limiting enzyme phosphofructokinase-1. Interestingly, *pfk-1*.*1(ola72)* suppressed the extended host survival during infection with *fadE2-* (Fig 5C; *p*-value 0.085). Thus, increased glycolytic flux mediates the extension in host survival observed during *fadE2-* infection.

Our metabolomic analysis also suggested a decrease in fatty acid metabolism during infection with *P*. *aeruginosa*, specifically long chain and very long chain fatty acid abundance (Fig 5D). In addition, lower levels of carnitine metabolites were observed during *P*. *aeruginosa* infection which are necessary for the transport of long-chain fatty acids into mitochondria for their eventual oxidation (S7B Fig). Once again, levels of these fatty acid and carnitine metabolites were largely restored to wild-type levels in the absence of FadE2 (Fig 5D and S7B Fig). To determine whether β-oxidation was involved with the increase in host survival observed during *fadE2-* infection, we genetically and chemically disabled carnitine palmitoyl transferase using the *cpt-5(gk5128)* loss of function mutant or through treatment with the inhibitor etomoxir, respectively. Interestingly, both *cpt-5(gk5128)* or etomoxir similarly suppressed the increase in host survival that occurred with *fadE2-* exposure (Fig 5E; *p*-value 0.404 and S8 Fig; *p*-value 0.873), indicating an involvement of β-oxidation.

Our metabolomic and transcriptomic analyses suggest that *P*. *aeruginosa* FadE2 represses host energy metabolism pathways that culminate with the TCA cycle in mitochondria. To examine whether increased energy metabolism mediates the increase in host survival observed during *fadE2-* infection, we used a viable reduction of function allele (*osa2*) in the succinyl-CoA ligase beta subunit gene *sucg-1* that we had isolated in an independent study (Amin et al. *in review*). SUCG-1 is the *C*. *elegans* homolog of human SUCGL2 which mediates the conversion of succinyl-CoA to succinate during the TCA cycle. Interestingly, *sucg-1(osa2)* mutants suppressed the increase in host survival observed during *fadE2-* infection (Fig 5F; *p*-value 0.784). Importantly, we observed no difference in host survival when *sucg-1(osa2)* mutants were infected with wild-type *P*. *aeruginosa*, indicating that *sucg-1(osa2)* animals are not simply succumbing to inherent metabolic distress (Fig 5F; *p*-value 0.934). Therefore, our data suggest *P*. *aeruginosa* FadE2 impairs host survival by reducing host energy metabolism. In the absence of FadE2, host energy production is increased resulting in ROS generation and activation of the UPR^mt^.

### Valine or leucine supplementation is sufficient to sustain UPR^mt^ activity and increase host survival during *P*. *aeruginosa* infection

**Fig 6:**
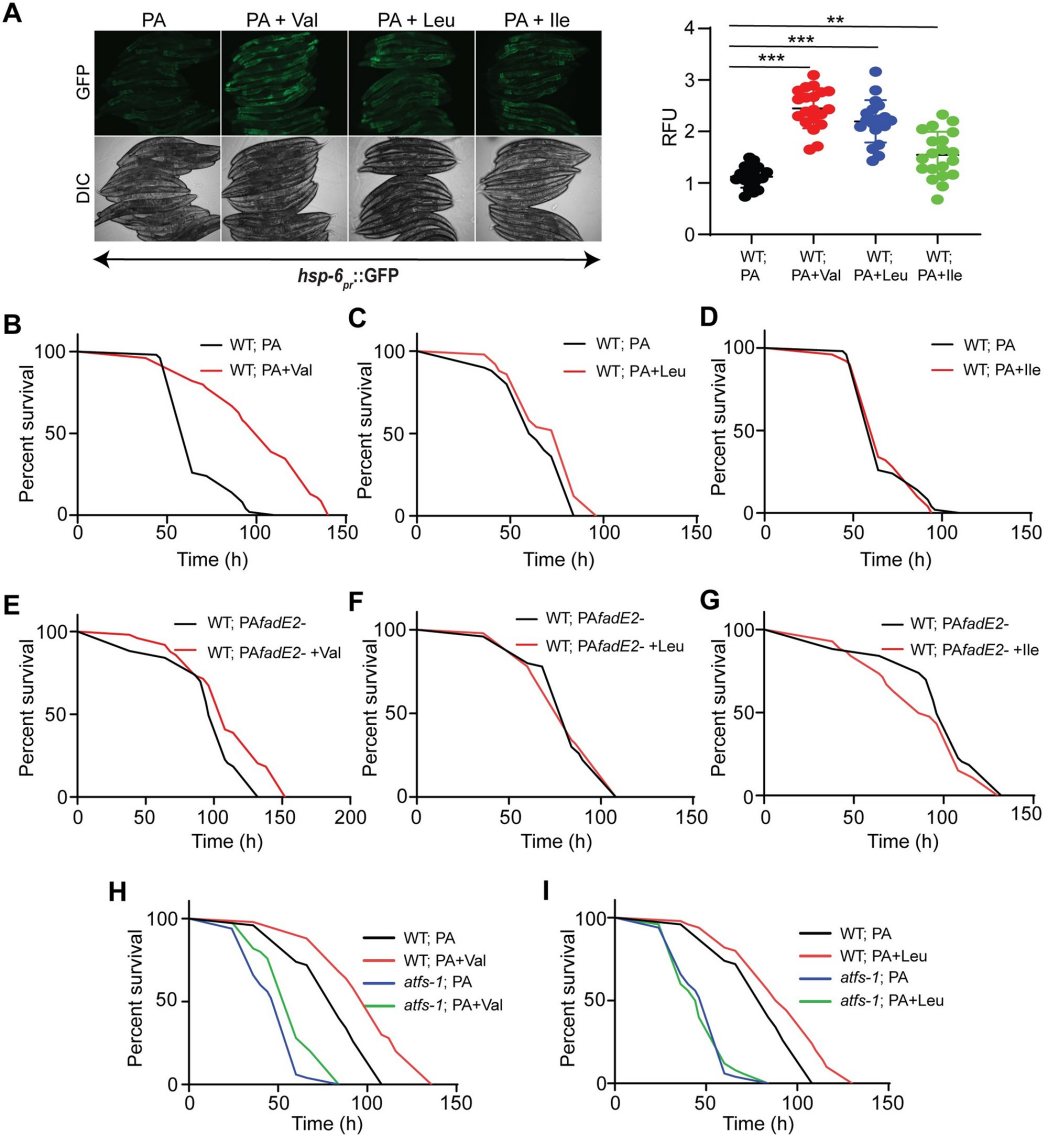
Valine or leucine supplementation is sufficient to restore the UPR^mt^ and protect the host during infection. (A) Expression of *hsp-6*_*pr*_::GFP in animals exposed to wild-type *P*. *aeruginosa* (PA) in the presence or absence of 5m M valine (Val), leucine (Leu), or isoleucine (Ile) for 48 hrs with quantifications of fluorescence. RFU: Relative Fluorescence Units. *** denotes p<0.001, ** denotes p<0.01, using Student’s *t*-test. (B-D) Survival of wild-type animals supplemented with 5 mM (B) valine, (C) leucine or (D) isoleucine infected with wild-type *P*. *aeruginosa*. (E-G) Survival of wild-type animals supplemented with 5 mM (E) valine, (F) leucine or (G) isoleucine infected with *fadE2-*. (H, I) Survival of wild-type or *atfs-1(cmh15)* animals supplemented with 5 mM (H) valine or (I) leucine infected with wild-type *P*. *aeruginosa*.

FadE2 showed substrate preference for the metabolic intermediates of valine and leucine catabolism. We hypothesized that *P*. *aeruginosa* FadE2 represses the UPR^mt^, host metabolism and host survival during infection by restricting the availability of valine and leucine catabolites. To test our hypothesis, we first asked whether we could counteract the repression of the UPR^mt^ during *P*. *aeruginosa* infection through simple supplementation of valine or leucine. Indeed, UPR^mt^ activity was maintained with valine or leucine supplementation, and to a lesser extent with isoleucine (Fig 6A). In contrast, supplementation of *E*. *coli* OP50 with valine, leucine, or isoleucine did not activate the UPR^mt^ (S9 Fig).

We next tested the effect of supplementing BCAAs on host survival during infection with *P*. *aeruginosa*. Remarkably, valine supplementation enhanced host survival during infection with wild-type *P*. *aeruginosa* (Fig 6B; *p*-value <0.0001) whereas only a mild increase was observed with leucine supplementation (Fig 6C; *p*-value 0.031) and no difference was observed with isoleucine supplementation (Fig 6D; *p*-value 0.438). Valine or leucine supplementation also increased the lifespan of animals fed standard *E*. *coli* OP50 (S10 Fig; *p*-values 0.002 and 0.003, respectively), despite a lack of UPR^mt^ activation. However, this in line with the pro-longevity benefits of BCAAs that were previously reported [25].

In contrast, a relatively smaller increase in host survival was observed during infection with *fadE2-* when animals were supplemented with valine (Fig 6E; *p*-value 0.002), whereas no difference was observed with leucine supplementation (Fig 6F; *p*-value 0.519). As expected, isoleucine had no effect on host survival during infection with *fadE2-* (Fig 6G; *p*-value 0.2). Since the increase in host survival by valine or leucine supplementation was not additive to the increase in host survival observed with *fadE2-* suggest they both use a common mechanism of action. Therefore, valine or leucine catabolite availability determines UPR^mt^ activity and host survival rates during infection.

Since valine and leucine supplementation maintained the activity of the UPR^mt^ during infection with wild-type *P*. *aeruginosa*, we also examined whether this protective pathway was required for the associated extension in host survival. Indeed, loss of ATFS-1 function suppressed the increase in host survival observed with valine (Fig 6H; *p*-value <0.0001) or leucine supplementation (Fig 6I; *p*-value <0.0001), rendering animals hypersensitive to infection.

We then examined whether glycolysis and β-oxidation mediated the extension in host survival with valine or leucine supplementation since both metabolic pathways were required during infection with *fadE2-*. Reducing glycolysis using the *pfk-1*.*1(ola72)* mutant suppressed the increase in host survival with valine (S11A Fig; *p*-value 0.102) or leucine supplementation (S11B Fig; *p*-value 0.321). Similarly, impaired β-oxidation through treatment with etomoxir or the *cpt-5(gk5128)* mutant yielded similar outcomes (S11C–S11F Fig; *p*-value ≥0.075). Since both glycolysis and β-oxidation mediated the beneficial effects afforded by valine or leucine, we next explored whether the increase in host survival with valine or leucine supplementation was dependent on increased energy metabolism using *sucg-1(osa2)* animals. Similar to what was observed during infection with *fadE2-*, *sucg-1(osa2)* completely suppressed the extension in host survival conferred by valine (S11G Fig; *p*-value 0.808) and leucine (S11H Fig; *p*-value 0.461) during infection with wild-type *P*. *aeruginosa*. Therefore, valine and leucine promote host survival during *P*. *aeruginosa* infection via pathways related to host energy metabolism and UPR^mt^ activation.

## Discussion

**Fig 7:**
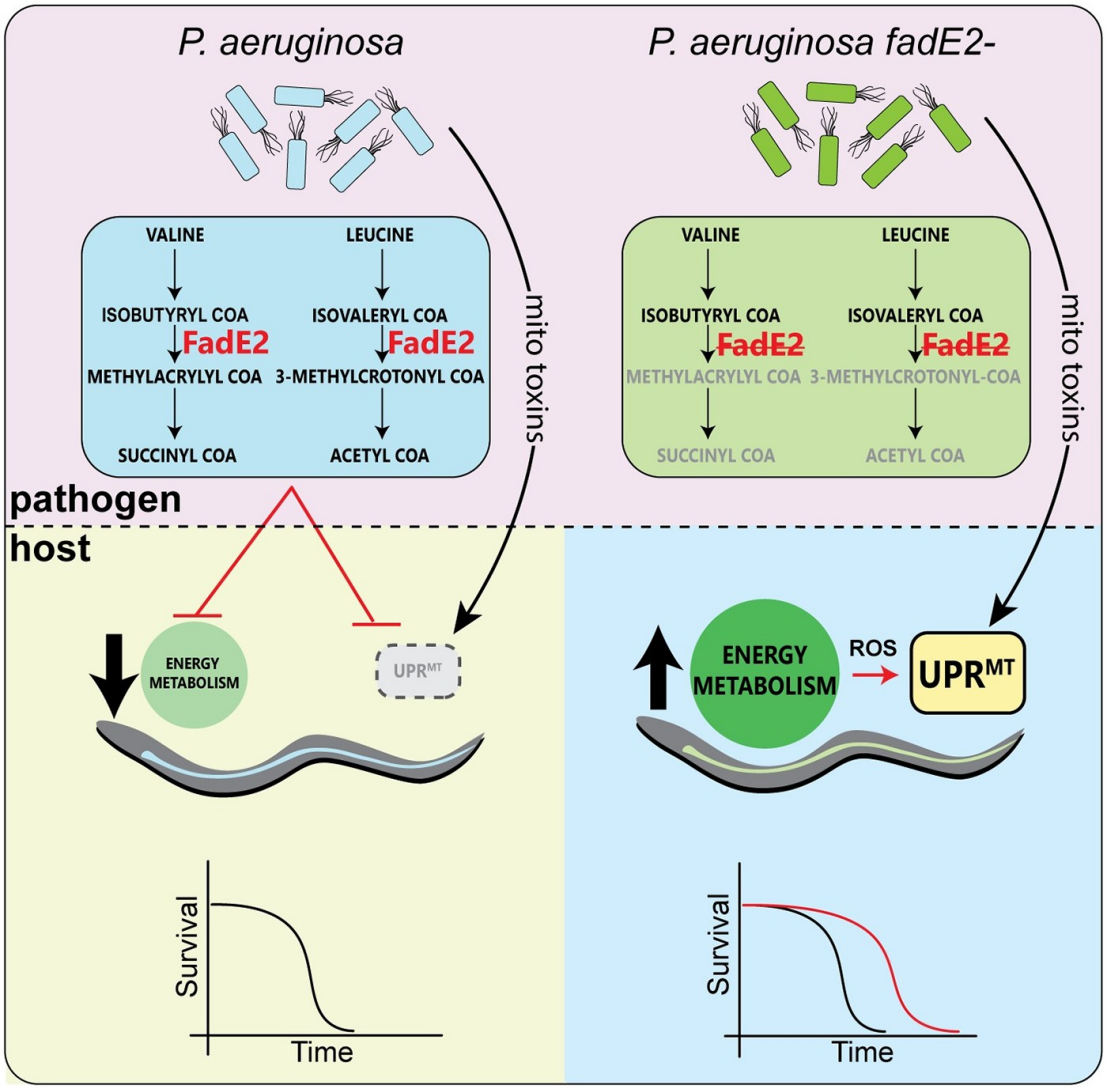
Model. During infection, *P*. *aeruginosa* FadE2 restricts valine and leucine catabolites which reduces host energy metabolic pathways and represses the UPR^mt^ through a presently undefined mechanism. However, loss of *P*. *aeruginosa* FadE2 results in accumulation of valine and leucine catabolites stimulating an increase in host energy metabolism and a restored ability to activate the UPR^mt^ that supports host survival. Consequently, the host relies on the activation of the UPR^mt^ to support mitochondrial recovery resulting from the stress caused by enhanced energy metabolism and by toxins produced by *P*. *aeruginosa* that target mitochondrial function.

We propose the following model explaining a mechanism used by *P*. *aeruginosa* to repress the UPR^mt^ during infection via the acyl-CoA dehydrogenase FadE2 (Fig 7). We show that FadE2 possesses substrate specificity for isobutyryl CoA and isovaleryl CoA, catabolites produced during the breakdown of the BCAAs valine and leucine, respectively. FadE2 activity during infection limits the availability of these catabolites for the *C*. *elegans* host which, through an as of yet unknown mechanism, hinders the activation of the UPR^mt^. We also show that the actions of FadE2 impair host energy pathways such as glycolysis, β-oxidation, and amino acid metabolism, all of which culminate with the TCA cycle. Consequently, loss of FadE2 results in a buildup of these catabolites in *P*. *aeruginosa*, resulting in a greater supply for the host which responds by restoring host energy metabolism and the ability to activate the UPR^mt^ through a mechanism that is currently not resolved. We favor a model in which loss of FadE2 allows the host to activate the UPR^mt^ in response to toxins produced by *P*. *aeruginosa* that target mitochondrial function (e.g. cyanide, pyocyanin etc.) in addition to the stress that is produced with increased mitochondrial respiration and the associated production of damaging ROS species. Together, both the increase in energy production and restored function of the UPR^mt^ promote host survival during infection.

The exact mechanism of how FadE2 affects UPR^mt^ activity during infection via changes in valine or leucine catabolite levels is presently not known. Recently, it was shown that the *C*. *elegans* bZIP transcription factor ZIP-3 is involved with the repression of the UPR^mt^ during infection with *P*. *aeruginosa* [15]. Here, *P*. *aeruginosa* exploits the repressive activities of this transcription factor on the UPR^mt^. The mechanism of how *P*. *aeruginosa* manipulates this host transcription factor are unclear. One possibility is that ZIP-3 mediates the repressive activities exerted by FadE2 on the UPR^mt^. Indeed, we have compared the genes negatively regulated by FadE2 and ZIP-3 and found modest overlap (S12 Fig), suggesting they may occur in the same pathway to some degree. Another possibility that might explain the repressive activities of FadE2 on the UPR^mt^ may relate to its ability to reduce host nutrient levels (e.g. amino acids, glucose) during *P*. *aeruginosa* infection. Accordingly, multiple host nutrient-sensing pathways [26] may be differentially regulated by FadE2 that may impact the activation of the UPR^mt^. The molecular mechanism of UPR^mt^ repression by FadE2 during *P*. *aeruginosa* infection is discernibly an active area of investigation.

Our model posits that increased energy metabolism drives host survival during infection with *P*. *aeruginosa*. Consistent with our model, increased energy metabolism has also been connected with improved host survival during infection in zebrafish. Here, increased supply of TCA cycle metabolites increased zebrafish survival during infection with *Vibrio alginolyticus* [27, 28]. Similar benefits of increased metabolism have been observed in other experimental scenarios outside of infection. For example, enhanced oxidation of fuel sources via the TCA cycle was shown to be necessary for the extension in animal longevity that is observed upon caloric restriction [29]. Also, hepatocytes have been shown to increase TCA cycle flux to promote cell survival during ischemia [30]. In addition, we also show that the host responds metabolically to the presence or absence of *P*. *aeruginosa* FadE2 in part through transcriptional re-wiring. The ability of animals to adapt to different metabolic demands through a transcriptional regulatory mechanism has also been observed in other contexts. For example, a transcriptional rewiring program occurs in *C*. *elegans* to support the breakdown of propionate in lieu of low vitamin B12 levels that acts as an enzyme cofactor in the catabolism of this metabolite that can result in toxicity if elevated [31, 32]. Also, during mouse embryonic development, a transcriptional rewiring in glucose metabolism occurs to support chorioallantoic branching [33].

Our data supports a model in which intermediates of valine and leucine catabolism reinforce host survival during infection. As essential amino acids, BCAAs mediate numerous cellular functions including protein synthesis, as well as glucose and lipid metabolism [34]. In addition, valine supplementation was recently shown to increase macrophage phagocytosis of multiple bacterial pathogens including *P*. *aeruginosa* using mouse models of infection [35]. Valine supplementation is thought to increase macrophage phagocytosis through activation of PI3K/Akt1 and also through the production of nitric oxide [35]. What remains unclear are the exclusive properties afforded by valine and leucine, but not isoleucine, that promote host survival during infection with *P*. *aeruginosa* in our *C*. *elegans* infection model. It is interesting that the CoA intermediates of valine and leucine are metabolized by FadE2, and that valine or leucine supplementation increased host survival. In contrast, FadE2 did not metabolize the CoA intermediate of isoleucine and consistently, isoleucine supplementation did not protect the host during infection. One possibility is that unique signaling pathways might be activated downstream of each of these essential amino acids. Indeed, leucine was shown to regulate the target of rapamycin complex 1 (TORC1), a pro-growth kinase, via acetylation of the TORC1 regulator Raptor using its final catabolic metabolite acetyl-CoA [36]. TORC1 was also shown to be activated via the cytoplasmic leucine sensor SESN2 [37, 38]. And, leucine has also been shown to increase insulin levels through allosteric activation of glutamate dehydrogenase [39]. With regard to metabolites related to valine catabolism, 3-HIB and beta-amino-isobutyric acid have been shown to induce their own signaling mechanisms [40]. Interestingly, 3-HIB is the only catabolite that lacks the CoA attachment, allowing the molecule to leave the mitochondrial matrix. Indeed, 3-HIB has been detected in plasma samples where it acts in paracrine signaling [40]. Based on the substrate preference of FadE2, it is likely that either isobutyryl-CoA and/or isovaleryl-CoA accumulation due to loss of FadE2 might stimulate the host to rewire its metabolism and allow activation of the UPR^mt^. However, we cannot exclude the possibility that derivatives of these metabolites may instead play some role.

During the infection process, *P*. *aeruginosa* can also be viewed as a food source for *C*. *elegans* which it digests to extract important nutrients (although this is only a partial digestion since live *P*. *aeruginosa* is able to colonize the intestinal tract of *C*. *elegans*). As such, it is possible that the actions of FadE2 restricts the supply of valine and leucine catabolites, thus resulting in a competition for nutrients between pathogen and host that dictates the potential for the UPR^mt^ to be activated. It is unknown whether a similar competition for nutrients would exist in the context of a *P*. *aeruginosa* infection in vertebrates. Nonetheless, it is tempting to speculate that *P*. *aeruginosa* FadE2 has a particular function during infection to restrict the supply of important metabolites. Indeed, *P*. *aeruginosa* FadE2 showed highest homology with acyl-CoA dehydrogenases from other pathogenic bacterial species and therefore may play an important role during infection of the host. Competition for nutrients critically determines the success of either the host or pathogen during infection since the site of infection can be a restrictive environment. For example, the host relies on certain amino acids during infection to mount an immune response [41]. From the perspective of the pathogen, specific amino acids have been linked with the expression of disease-promoting virulence factors. For example, L-glutamine levels dictate the expression of virulence factors in *Listeria monocytogenes* within macrophages [42]. Consequently, reducing L-glutamine availability restricts expression of *L*. *monocytogenes* virulence factors and increases health of the host.

Together, our study demonstrates that a *P*. *aeruginosa* metabolic pathway negatively impacts both host energy metabolism and the activation of the UPR^mt^. This occurs presumably due to an ability of *P*. *aeruginosa* to successfully outcompete the host for specific nutrients related to the catabolism of valine or leucine. The molecular mechanisms linking these catabolites to host energy pathways and mitochondrial stress signaling are still unresolved and therefore will be an exciting area of future research.

## Material and methods

### *C*. *elegans* strains

*C*. *elegans* was maintained on standard Nematode Growth Media (NGM) according to previously described methods [43]. *C*. *elegans* strains were provided by the Caenorhabditis Genetics Center: N2 Bristol; SJ4100 *zcIs13*[*hsp-6*::GFP], SJ4005 *zcIs4*[*hsp-4*::GFP], *pfk-1*.*1(ola72)*, *cpt-5(gk5128* [loxP+P*myo-2*::GFP::*unc-54* 3’UTR + P*rps-27*::neoR::*unc-54* 3’UTR + loxP]*)* [44], AU133 *agIs17*[*irg-1*::GFP][22]. UTA37 *sucg-1(osa2)* was isolated in a forward genetics screen using ethyl methanesulfonate (Amin et al.; *in revision*). *atfs-1(cmh15)* was a gift from Dr. Cole M. Haynes (University of Massachusetts Medical School). All mutant animals were backcrossed to N2 at least four times prior to use.

### Pathogen infection assays

*P*. *aeruginosa* plates were prepared using overnight cultures that were incubated for 18 hrs at 37°C before seeding 15 μL of bacterial culture onto NGM plates. The plates were incubated at room temperature for 24 hrs followed by transferring to 37°C for 24 hrs. The plates were then incubated at 25°C for 3 hrs before transferring the *C*. *elegans* animals for the infection assay. Wild-type animals were synchronized and grown at 16°C for 72 hrs until the fourth larval stage before transferring to *P*. *aeruginosa* plates. Survival assays was carried out at 25°C for the indicated time intervals. Statistical analysis was calculated using GraphPad Prism version 8 (GraphPad Software, San Diego, California, USA) where *p*-values were generated by the log-rank (Mantel-Cox) test. Statistical tests were performed for each strain/condition compared to the wild-type control. For all cases, *p*-values of <0.05 were considered significant. All statistical values are presented in S2 Table.

### Microscopy

Worms were imaged using a Zeiss AxioCam MRm mounted on a Zeiss Imager Z2 microscope. Same exposure time was used for each experiment. Total fluorescence was quantified using ImageJ and the relative intensity between worm strains were compared using a one-way ANOVA.

### *C*. *elegans* pathogen avoidance

The bacterial lawns were prepared as described earlier. Forty L4 worms for each condition were transferred outside the bacterial lawns, and the numbers of animals on and off the lawns were counted for each experiment after 18 hrs. Three 3.5-cm plates were used per trial in every experiment. The percent occupancy was calculated as (N_on_/N_total_) X 100. At least three independent experiments were performed.

### *P*. *aeruginosa fadE2* rescue in *P*. *aeruginosa ∆fadE2*

The 1230 bp *fadE2* ORF along with 500 bp upstream sequence was amplified form *P*. *aeruginosa* wild-type genomic DNA using primers 5’-TTTGCTAGCCGCTTCGGTGAGGTCGGTCAT-3’ and 5’-TTTAAGCTTTCAGCGGCTGCTGCGCAGCAT-3’ and cloned into *NheI* and *HindIII* sites of the plasmid pUCP20T, thus replacing the original IPTG promoter of the plasmid with the upstream promoter region of *fadE2*. The resulting plasmid, pUCP20T::fadE2_pr_::fadE2, was transformed into *P*. *aeruginosa fadE2-* using electroporation. The transformants were selected on plates containing gentamicin (50 μg/ml) and carbenicillin (300 μg/ml).

### Oxygen consumption rate (OCR) assay

The OCR assay was performed according to [45]. The MitoXpress Xtra oxygen consumption assay kit (Agilent, USA) was used to calculate the OCR from 100 to 150 worms. Approximately 100–150 worms were recovered from NGM plates and washed three times in S-basal to remove excess bacteria. Worms were then transferred to wells of a 96-well plate in a final sample volume of 90 μl. The oxygen probe was then added to each sample at a volume of 10 μl. The 96-well plates were then sealed with two drops of mineral oil and sample wells were immediately read on Synergy Neo 2 plate reader using Gen5 software (BioTek, Wisnooski, VT, USA) in a time-resolved fluorescence mode with 380 nm excitation and 650 nm emission filters. Data were collected from 0 hr to 1 hr at 25 °C. To eliminate background oxygen consumption, controls (in triplicates) with S-basal and S-basal plus oxygen probe, but no worms, were included in each respiration assay. The measured time profiles of fluorescence from each sample were normalized to the signal at time zero to obtain normalized intensity, and they were analyzed to determine the slope of each sample, the slopes, which reflect the oxygen consumption rates by nematodes, were determined by selecting the linear portion of the signal profile and applying the linear regression according to the probe’s manufacturer’s instructions. Calculated slopes were used to determine the respiration rates in each sample. The relative respiration rates of *C*. *elegans* was calculated as follows: R = (Ss–Sn)/Sp, where Ss, Sn and Sp is the slope of the sample, the negative control and the positive control. Each condition was analyzed in three replicates on the 96-well plates. The OCR experiment was repeated four times.

### Measurement of ATP production

ATP was quantified using a bioluminescence ATP measurement kit (Thermo Fisher Scientific, Waltham, MA, USA). Worms were collected from NGM plates and washed three times in S-basal to remove bacteria and frozen into liquid nitrogen. Before starting the analysis, the samples were put into a heat block at 95 °C for 15 min, then placed in ice for 5 min. Next, samples were spun down at 14,000 x *g* for 10 min at 4 °C and the supernatant was used to measure ATP. 10 μl of each sample in duplicates were transferred into 96-well plates. The ATP assay solution was prepared according to the manufacturer’s instructions. 90 μl of the assay solution was then added to each sample. Next, the sample wells were read on Synergy Neo 2 plate reader using Gen5 software (BioTek, Wisnooski, VT, USA) with a luminometer filter. An ATP standard curve was generated and the ATP concentration for each sample was calculated based on the standard curve.

### Protein oxidation measurement by OxyBlot

The level of protein oxidation was measured with the OxyBlot protein oxidation detection kit (Millipore-Sigma, Burlington, MA, USA). Worms were collected from each condition, washed three times with S-basal and concentrated to 10 μl lysis buffer and frozen to -80 °C for storage. Worms were homogenized with a TissueLyser II (Qiagen, Germantown, MD, USA). The DNP reaction mixture was made by adding 15 μg protein for each sample adjusted in 7 μl, 3 μl of 15% SDS and 10 μl of DNP solution. The mixture was kept at room temperature for 15 min, after which 7.5 μl Neutralization buffer was added. Samples (27.5 μl) were run in 10% SDS-PAGE gels, transferred into nitrocellulose (Bio-Rad, Hercules, California, USA) and blocked with 5% non-fat milk for 1h. After washing, the membrane was incubated with the first antibody (1:150) overnight at 4 °C and then for 1h with the secondary antibody (1:300) at room temperature. Membranes were incubated with ECL plus detection reagent (Bio-Rad) and scanned using Chemiluminescent scanner (Bio-Rad). Band densities were analyzed using ImageJ. Membranes were then incubated with 15% hydrogen peroxide for 30 min at room temperature and treated with actin antibody to derive a density value of actin for each lane, with which OxyBlot value was normalized.

### Quantification of mitochondrial membrane potential

Young adult worms were infected with *P*. *aeruginosa* for 12, 24 or 48 hrs at 25 °C on NGM plates that were pre-treated with 500 nm tetramethylrhodamine ethyl ester perchlorate (TMRE). To remove excessive dye from gut, worms were transferred to NGM agar plates with appropriate *P*. *aeruginosa* backgrounds for an additional 1 hr. Photographs were taken immediately.

### FadE2 protein sequence analysis

The FadE2 protein sequence was blasted against NCBI non-redundant protein database using BLASTP and the top ten hits were identified. Protein sequence of the top 10 hits were gathered from Uniprot and multiple sequence alignment of those protein sequences was performed through the ClustalW online alignment tool. The resulting alignment file was imported into Bio-Edit to construct the final alignment figure. For the generation of phylogenetic tree, the same alignment file was imported into MEGA 7.0 software and the tree generated using the Neighbor-joining method using 1000 bootstraps.

### Bacterial growth on fatty acids and amino acids

Fatty acids (butyric acid, C4: 0; N-caproic acid, C6: 0; caprylic acid, C8: 0; capric acid, C10: 0; lauric acid, C12: 0; myristic acid, C14: 0; palmitic acid, C16: 0 and oleic acid, C18:1) stock solutions at 3% (w/v) were made with equimolar KOH and 1% Brij-58 [poly(oxyethylene) cetyl ether]. Growth curves were performed in 1× M9 (containing 0.5 mM MgCl_2_ and 0.02 mM CaCl_2_)+1% Brij-58 supplemented with 20 mM glucose or 0.2% of different fatty acids as sole carbon source.

For BCAAs, 5% stock solution was made for valine and 2% for leucine and isoleucine. Growth curves were performed in 1x M9 (containing 0.5 mM MgCl_2_ and 0.02 mM CaCl_2_) with 0.2% amino acids as sole carbon source. Bacterial cultures were grown overnight in LB medium and then washed with PBS three times to get rid of media. Cultures of bacteria were diluted to an initial OD_600_ of 0.01 for fatty acid or amino acid media. Unless indicated otherwise, the cultures were grown at 37 °C with a shaking speed of 250 rpm.

To calculate the specific growth rate, we used OD_600_ values obtained 8–44h after inoculation. *p*-values were obtained from specific growth rates in three independent experiments using the Student’s t-test.

### Recombinant expression and purification of FadE2 protein in *E*. *coli*

For recombinant expression of FadE2 protein in *E*. *coli*, the 1230 bp coding sequence was amplified from *P*. *aeruginosa* wild-type genomic DNA using primers GCAGCCATATGATGGATTTCGCCTATTCCCCCAA and ATCATCTCGAGTTAGCGGCTGCTGCGCAGCATCT and ligated into *NdeI* and *XhoI* sites of the expression vector pET28a(+) to yield pET28a::fadE2. The recombinant strain, *E*. *coli fadE2* was grown in LB medium supplemented with ampicillin (50 μg/ml). Cultures were grown at 37 °C with shaking at 280 rpm until optical density reached OD_600_ of 0.5, at which point cells were induced with 0.1mM IPTG at 25 °C for 6 hrs. After induction, cells were harvested and FadE2 purified using a His-Trap column procedure described previously [46]. Fractions were collected and analyzed with 10% SDS-PAGE under denaturing conditions.

### Analysis of acyl-CoA dehydrogenase activity using purified FadE2 protein

The DCPIP method was used to determine the ACAD activity of FadE2 [47]. In brief, the 200 μl assay mixture contained 50 mM HEPES-KOH buffer (pH 8.0), 1mM KCN, 1mM salicylhdroxamic acid, 50 μM FAD, 100 μ/ml DCPIP, 100 μg/ml PMS 50 μM of each of the fatty-acyl CoA substrates, 50 μl of purified FadE2. The reaction was started by the addition of PMS and its progress was followed by measuring the decrease in absorbance at 600 nm at 25 °C. An absorption coefficient of DCPIP 13100 M^-1^ cm^-1^ was used to calculate rates. Reaction rates were calculated from the initial linear portions of the absorbance versus time plots.

### *C*. *elegans* metabolic profiling

Metabolic analyses were conducted in Metabolon as previously described [48]. Briefly, nematode pellet samples were homogenized and subjected to methanol extraction then split into aliquots by ultrahigh performance liquid chromatography/ mass spectrometry (UHPLC/MS) in the positive (two methods) and negative (two methods) mode. Metabolites were then identified by automated comparison of ion features to a reference library of chemical standards followed by visual inspection for quality control (as previously described, [49]). For statistical analyses and data display, any missing values are assumed to be below the limits of detection; these values were imputed with the compound minimum (minimum value imputation). Bradford protein measurements were conducted in parallel to normalized data to amount extracted. For generation of graphs, *p*-value ≤ 0.05 is considered significant. An estimate of false discovery rate (Q-value) is also calculated to take into account the multiple comparisons that normally occurs in metabolomics-based studies, with Q < 0.05 used an indication of high confidence in result.

### RNAseq, quality analysis, mapping, assembly and differential expression

The total RNA was extracted using Trizol reagent according to the manufacturer’s instructions and purified using RNA purification kit. RNA purity was checked using the Nanodrop Spectrophotometer. The RNA integrity was assessed using the Bioanalyzer 2100 system.

Sequencing libraries were generated in Novogene Inc. (CA, USA) using the NEBNext Ultra RNA Library Kit from Illumina (NEB, Ipswich, MA, USA), following manufacturer’s protocol. The prepared DNA library was sequenced on an Illumina Hiseq 4000 according to the manufacturer’s instructions for paired-end 150-bp reads. Clean data were then obtained by removing reads containing the adapter, reads containing poly-N and low-quality reads (<Q30) from the raw data using the program Trimmomatic [50]. Cleans reads were aligned to the *C*. *elegans* reference genome using TopHat v.2.0.9 (Trapnell et al., 2012). The mapped reads from each sample was assembled using Cufflinks v.2.1.1 [51]. HTSeq v.0.6.1 [52] was used to count the number of reads mapped to each gene. In addition, the reads per kilobase million (RPKM) of each gene was calculated based on the length of the gene and the number of reads mapped to it. Pairwise differential expression analysis was performed using *DESeq*2 R package (v.1.10.1) [53]. Genes with adjusted *p*-value ≤0.05 were considered to be differentially expressed. The raw sequencing data are available from the NCBI SRA database and are archived under the accession number PRJNA664413. Heat maps were generated using R Studio.

### *P*. *aeruginosa* virulence assays

#### Swarming motility assay

Motility assay was performed to assess the ability of bacteria to swim. In this test, each strain is stabbed into motility agar (LB plates containing 0.3% agar) and the plates were incubated at 37 °C for 48 hrs. As nutrients deplete, cells swim outwards forming circles of cells, or ‘swarms’. Cells defective in flagellar-mediated motility do not form swarms but remain clustered in the stabbing area.

#### Twitching motility assay

Twitching motility assay was performed to investigate the movement by some type IV pili which is independent of flagella. For this, the bacterial strains are stabbed into thin LB plates containing 1.5% agar with a toothpick and plates are incubated at 37 °C for 72 hrs. Strains proficient for type IV pili-mediated twitching motility form a hazy zone of growth at the interface between the agar and the petri plate.

#### Biofilm formation assay

A static biofilm assay was conducted with polypropylene tube. Bacterial strains were grown overnight at 37 °C under constant rotation. The resulting suspension was diluted with LB broth to give an optical density at 600 nm (OD_600_) = 0.01. 1 ml of diluted culture was transferred into a polypropylene tube. The plate was incubated at 30 °C for 48h. Planktonic cells were carefully removed by washing tubes with sterile double distilled water three times, and the plate was air dried. A 2 ml volume of 0.1% crystal violet (w/v) in 20% (v/v) ethanol/water was added into each tube to stain biofilm cells and incubated at room temperature for 1 hr in dark. Unbound dye was removed by washing with water three times. The crystal violet bound to biofilm was dissolved in 1 ml of 100% ethanol. The crystal violet amount for biofilm cells was measured at 595 nm. The relative biofilm forming ability was calculated by dividing A_595_ nm by the total crystal violet by the A_600_ nm for the cell density.

#### Protease assay

Overnight grown bacterial culture was used to aseptically streak a single line onto freshly prepared Milk agar plates (10% skim milk, 2% agar, 0.5% peptone, pH 7.2). The plates were incubated overnight in an inverted position for 48 hrs at 37 °C. Milk agar plates were examined for the presence or absence of clear zone area or zone of proteolysis, surrounding the growth of each of the strains.

#### Elastase and Rhamnolipid assays

Elastase and Rhamnolipid assays were done following the protocol from [54]. In brief, bacterial strains were grown overnight at LB broth at 37 °C to reach stationary phase. The culture was then diluted with LB broth to yield an initial OD_600_ of 0.01 and then grown under shaking conditions at 37 °C until they reach mid-log phase. Using an inoculation loop, bacterial culture was streaked as a single line on elastin or rhamnolipid plates and incubated for 48 hrs at 37 °C. Plates were examined for the appearance of clearings surrounding the culture.

#### Pyocyanin assay

Freshly grown bacterial colony was used to inoculate 5 ml LB with or without gentamicin for wild-type or *fadE2* mutants, respectively and grown at 37 °C for 40 hrs. The bacteria was pelleted by centrifugation and the amount of blue pigment pyocyanin was measure in supernatants at 690 nm. The relative pyocyanin excretion ability was calculated by dividing A_960_ nm by A_600_ nm for the cell density.

#### LPS determination

Determination of the Kdo sugars was used to estimate the LPS concentration in the sample using the TBA method as follows: Bacteria were grown on 35 mm NGM plates for 24 hrs at 37°C, collected from agar plates and resuspended in 1 ml of sterile water. 50 μl of sample (OD600 = 0.2) or standard (0 to 20 μg/ml) were mixed with 50 μl of 0.5 M H_2_SO_4_. Samples were boiled for 8 min to release the Kdo sugars and cooled at RT for 10 min. 50 μl of 0.1 M periodic acid was added, vortexed and incubated for 10 min. Next, 200 μl of 0.2 M sodium arsenite in 0.5 M HCl was added and vortexed, followed by the addition of 800 μl of freshly prepared 0.6% (w/v) thiobarbituric acid (TBA). After vortexing, samples and standards were boiled for 10 min, cooled at RT for 40 min and were split in two 575 μl portions. 750 μl of n-butanol equilibrated with 0.5 M HCl was added to each tube, followed my vortexing and spinning at 12000 *x* g for 6 min. The organic phases were recovered and combined into a cuvette and the absorbance was immediately taken at 552- and 509 nm. The 509 nm readings were subtracted from 552 nm readings for the standards to get a linear standard curve that was used to generate the concentration of unknowns.

#### Cyanide quantification

Cyanide production was quantified as described previously [13]. Strains were grown on 35 mm NGM plates for 24 h at 37°C and then enclosed without lids in individual sealed chambers which also contained 1 ml reservoir of 4 M NaOH (in an inverted 35 mm plate lid). All chambers were incubated at 25°C for 4 hrs, the NaOH was collected and diluted to 0.09M NaOH to bring the concentration within the detectable range (0–10 μM). The cyanide in the sample was quantified by comparison with standards of KCN in 0.09M NaOH: 105 μl aliquots of samples or KCN standards were mixed with 350 μl aliquots of freshly prepared 1:1 mixture of 0.1M *o*-dinitrobenzene and 0.2 M *p*-nitrobenzaldehyde (both in ethylene-glycol monoethyl ether). After incubation of 30 min at 22°C, OD578 was measured. Total protein was determined by collecting bacteria from agar plates and resuspending the cells in 1 ml of 0.85% NaCl. After centrifugation, the cells were lysed and protein precipitated in 5% trichloroacetic acid. Protein pellets were resuspended in 1 ml of 50 mM KH_2_PO_4_ and the total amount of protein was determined in Bio-rad protein measurement reagent.

## Supporting information

S1 Fig Loss of *P*. *aeruginosa* FadE2 enhances UPR^mt^ activity during infection. Quantification of fluorescence from *hsp-6*_*pr*_::GFP animals exposed to *E*. *coli* OP50, wild-type *P*. *aeruginosa* (PA) or *fadE2-* for 24 hrs. RFU: Relative Fluorescence Units. Shown is the mean ± SEM (n≥20 worms). *** denotes p<0.001 using Student’s *t*-test.
(TIF)

S2 Fig *fadE2-* does not affect *C*. *elegans* avoidance behavior. (A) Photomicrographs of wild-type animals grown in the presence of *E*. *coli*, wild-type *P*. *aeruginosa* (PA), or *fadE2-*. Scale bar is 1 mm. bac = bacteria, arrows point to position of *C*. *elegans* relevant to bacterial lawn edge. (B) Quantification of avoidance behavior of wild-type animals grown in the presence of *E*. *coli*, wild-type *P*. *aeruginosa* (PA), or *fadE2-*. Shown is the mean ± SEM (n≥20 worms). *ns* denotes no significance using Student’s *t*-test.
(TIF)

S3 Fig Glutathione metabolism decreases during infection with *P*. *aeruginosa* in a FadE2-dependent manner. Quantification of metabolites related to glutathione biosynthesis metabolism by mass spectrometry using extracts of wild-type animals exposed to *E*. *coli* OP50, wild-type *P*. *aeruginosa* (PA) or *fadE2-* following 24 or 48 hrs. Shown is the mean ± SEM (n≥4). See S1 Table for statistics.
(TIF)

S4 Fig *P*. *aeruginosa fadE2-* does not show evidence of impaired virulence. (A) Growth curves of wild-type *P*. *aeruginosa* (PA) or *fadE2-* in LB medium. (B) Growth curves of wild-type *P*. *aeruginosa* (PA) or *fadE2-* in NGM. (C) Photomicrographs and quantification of biofilm formation in wild-type *P*. *aeruginosa* (PA) or *fadE2-*. Shown is the mean ± SEM (n = 3). (D) Photomicrographs and quantification of swarming motility in wild-type *P*. *aeruginosa* (PA) or *fadE2-*. Shown is the mean ± SEM (n = 3). (E) Photomicrographs and quantification of twitching motility in wild-type *P*. *aeruginosa* (PA) or *fadE2-*. Shown is the mean ± SEM (n = 3). (F) Quantification of LPS levels in wild-type *P*. *aeruginosa* (PA) or *fadE2-*. Shown is the mean ± SEM (n = 3). (G-I) Photomicrographs showing production of (G) proteases, (H) rhamnolipids, and (I) elastase in wild-type *P*. *aeruginosa* (PA) or *fadE2-*. (J) Quantification of cyanide levels in wild-type *P*. *aeruginosa* (PA) or *fadE2-*. Shown is the mean ± SEM (n = 3). (K) Quantification of pyocyanin levels in wild-type *P*. *aeruginosa* (PA) or *fadE2-*. Shown is the mean ± SEM (n = 3). (C, D, E, F, J, K) *ns* denotes no significance using Student’s *t*-test.
(TIF)

S5 Fig Phenotypes associated with *fadE2-* can be rescued. (A) *hsp-6*_*pr*_::GFP animals grown in the presence of wild-type *P*. *aeruginosa* (PA), *fadE2-*, or *fadE2-* expressing a FadE2 rescue plasmid for 48 hrs. (B) Survival of wild-type animals during infection with wild-type *P*. *aeruginosa* (PA), *fadE2-*, or *fadE2-* expressing the FadE2 rescue plasmid. See S2 Table for survival assay statistics.
(TIF)

S6 Fig Acyl-CoA dehydrogenases mediate fatty acid and BCAA catabolism. Schematic overview of the role of acyl-CoA dehydrogenases (ACAD) in fatty acid and BCAA catabolism.
(TIF)

S7 Fig Gamma-glutamyl amino acids and carnitine metabolism decrease during infection with *P*. *aeruginosa* in a FadE2-dependent manner. Quantification of metabolites related to (A) gamma-glutamyl amino acids, (B) carnitine metabolism by mass spectrometry using extracts of wild-type animals infected with wild-type *P*. *aeruginosa* (PA) or *fadE2-* following 24 or 48 hrs. Shown is the mean ± SEM (n≥4).
(TIF)

S8 Fig Carnitine metabolism is required for the increased survival of animals infected with *fadE2-*. Survival of wild-type animals treated with 50 μM etomoxir (Eto) and infected with wild-type *P*. *aeruginosa* (PA) or *fadE2-*. See S2 Table for statistics.
(TIF)

S9 Fig Valine, leucine, or isoleucine supplementation does not activate the UPR^mt^ upon exposure to *E*. *coli* OP50. Photomicrographs and quantifications of *hsp-6*_*pr*_::GFP fluorescence for wild-type animals fed *E*. *coli* OP50 and supplemented with valine, leucine, or isoleucine. RFU: Relative Fluorescence Units. Shown is the mean ± SEM (n≥20 worms). Scale bar is 100 μm for all images. *ns* denotes no significance, *** denotes p<0.001, *denotes p<0.05 using Student’s *t*-test.
(TIF)

S10 Fig Valine or leucine supplementation increases the lifespan of *C*. *elegans* fed *E*. *coli* OP50. (A, B) Lifespans of wild-type animals supplemented with 5 mM (A) valine or (B) leucine fed a diet of *E*. *coli* OP50.
(TIF)

S11 Fig Increased host survival during *P*. *aeruginosa* infection with valine and leucine supplementation depends on host energy metabolism pathways. (A, B) Survival of *pfk-1*.*1(ola72)* animals infected with wild-type *P*. *aeruginosa* (PA) and supplemented with 5 mM (A) valine or (B) leucine. (C, D) Survival of wild-type animals treated with 50 μM etomoxir (Eto) and supplemented with 5 mM (C) valine or (D) leucine and infected with wild-type *P*. *aeruginosa* (PA). (E, F) Survival of *cpt-5(gk5128)* animals infected with wild-type *P*. *aeruginosa* (PA) and supplemented with 5 mM (E) valine or (F) leucine. (G, H) Survival of *sucg-1(osa2)* animals infected with wild-type *P*. *aeruginosa* (PA) and supplemented with 5 mM (G) valine or (H) leucine.
(TIF)

S12 Fig Modest overlap in the number of genes negatively regulated by *P*. *aeruginosa* FadE2 and *C*. *elegans* ZIP-3. Venn diagram demonstrating the number of commons genes that are negatively regulated by *P*. *aeruginosa* FadE2 (see S3 Table) and ZIP-3 (see reference [15]).
(TIF)

S1 Table Metabolomic analysis of wild-type animals exposed to *E*. *coli* OP50, wild-type *P*. *aeruginosa* or *fadE2-* for the defined period. (XLSX)

S2 Table Statistical analysis for all animal survival assays. (XLSX)

S3 Table RNA sequencing analysis of wild-type animals exposed to *E*. *coli* OP50, wild-type *P*. *aeruginosa* or *fadE2-* for the defined period. (XLSX)
