# Supplementary figures and images for: A pathogen branched-chain amino acid catabolic pathway subverts host survival by impairing energy metabolism and the mitochondrial UPR
Source: PLoS Pathog. 2020 Sep 30;16(9):e1008918. doi: 10.1371/journal.ppat.1008918 (PMC7549759; doi:10.1371/journal.ppat.1008918)

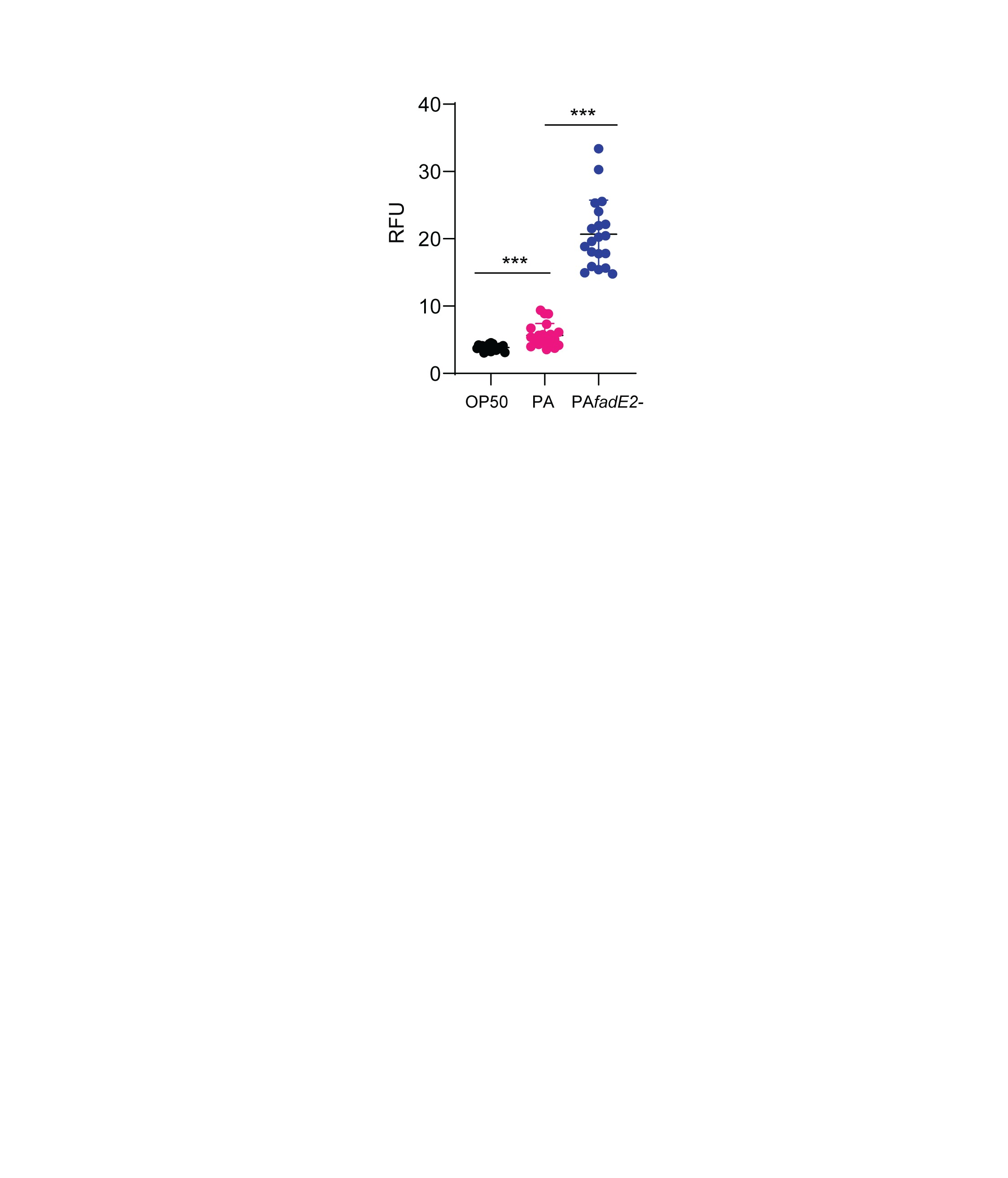

Supplement: S1 Fig — Quantification of fluorescence from hsp-6pr::GFP animals exposed to E. coli OP50, wild-type P. aeruginosa (PA) or fadE2- for 24 hrs. RFU: Relative Fluorescence Units. Shown is the mean ± SEM (n≥20 worms). *** denotes p<0.001 using Student’s t-test. (TIF) [file ppat.1008918.s001.tif]

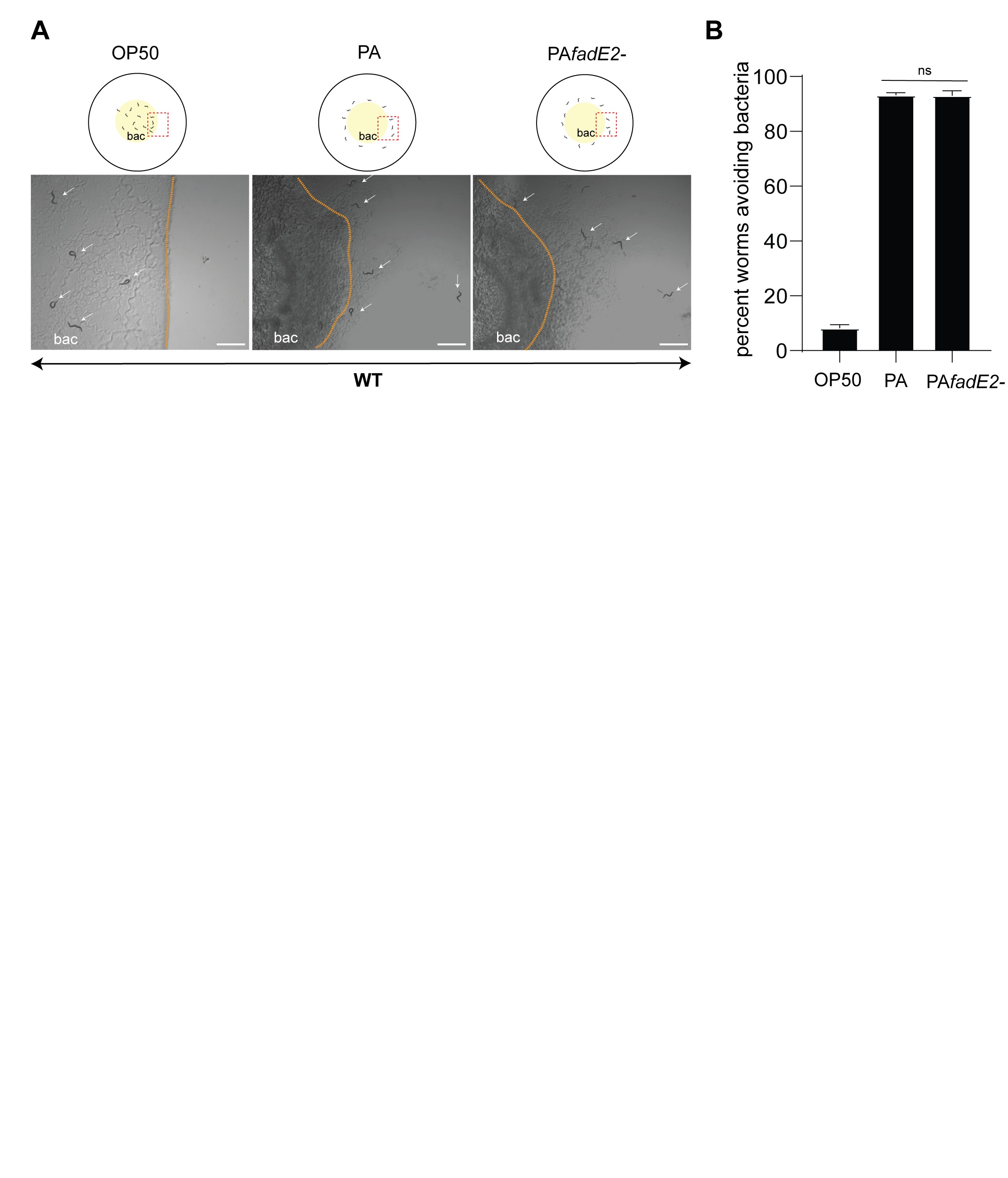

Supplement: S2 Fig — (A) Photomicrographs of wild-type animals grown in the presence of E. coli, wild-type P. aeruginosa (PA), or fadE2-. Scale bar is 1 mm. bac = bacteria, arrows point to position of C. elegans relevant to bacterial lawn edge. (B) Quantification of avoidance behavior of wild-type animals grown in the presence of E. coli, wild-type P. aeruginosa (PA), or fadE2-. Shown is the mean ± SEM (n≥20 worms). ns denotes no significance using Student’s t-test. (TIF) [file ppat.1008918.s002.tif]

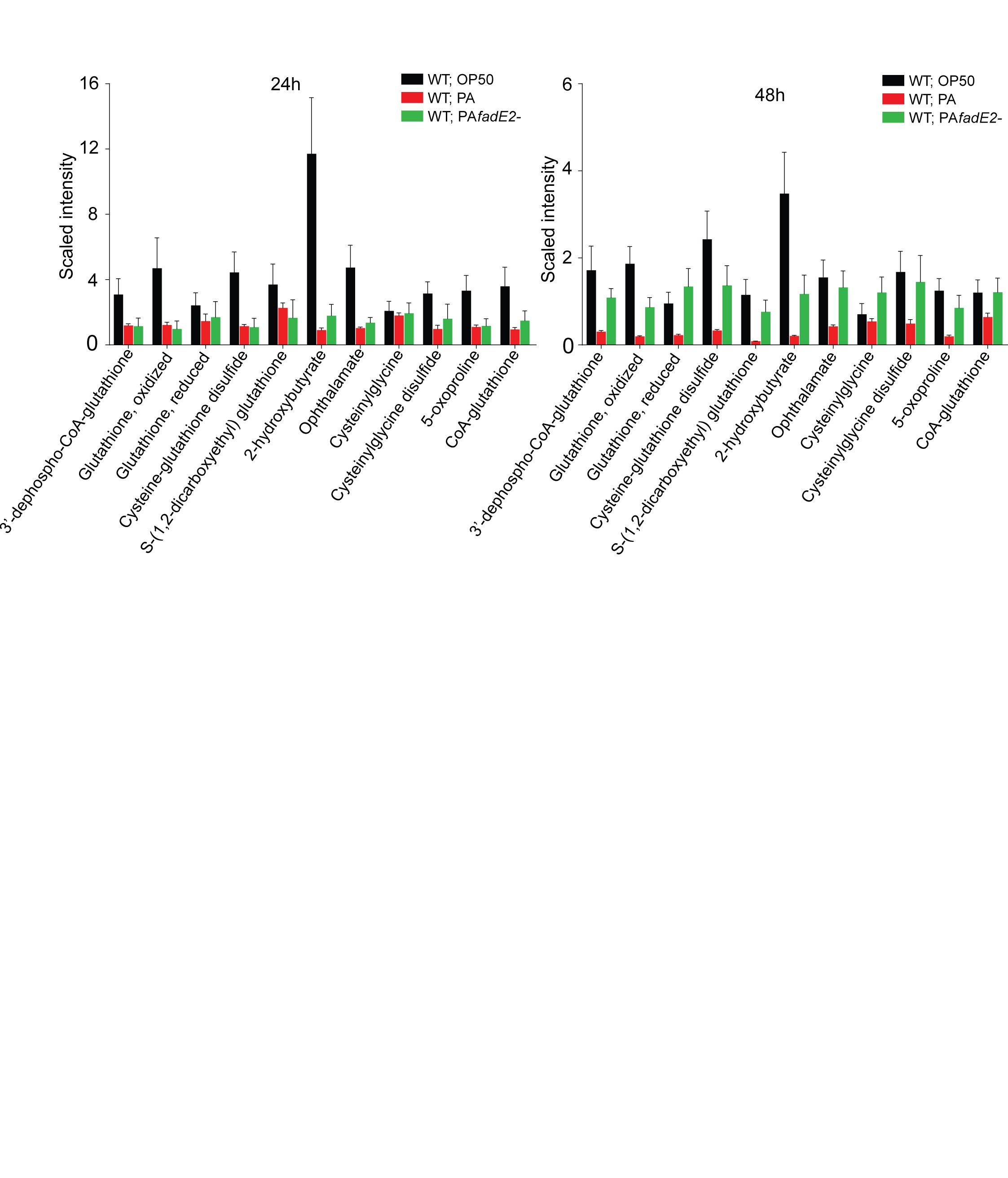

Supplement: S3 Fig — Quantification of metabolites related to glutathione biosynthesis metabolism by mass spectrometry using extracts of wild-type animals exposed to E. coli OP50, wild-type P. aeruginosa (PA) or fadE2- following 24 or 48 hrs. Shown is the mean ± SEM (n≥4). See S1 Table for statistics. (TIF) [file ppat.1008918.s003.tif]

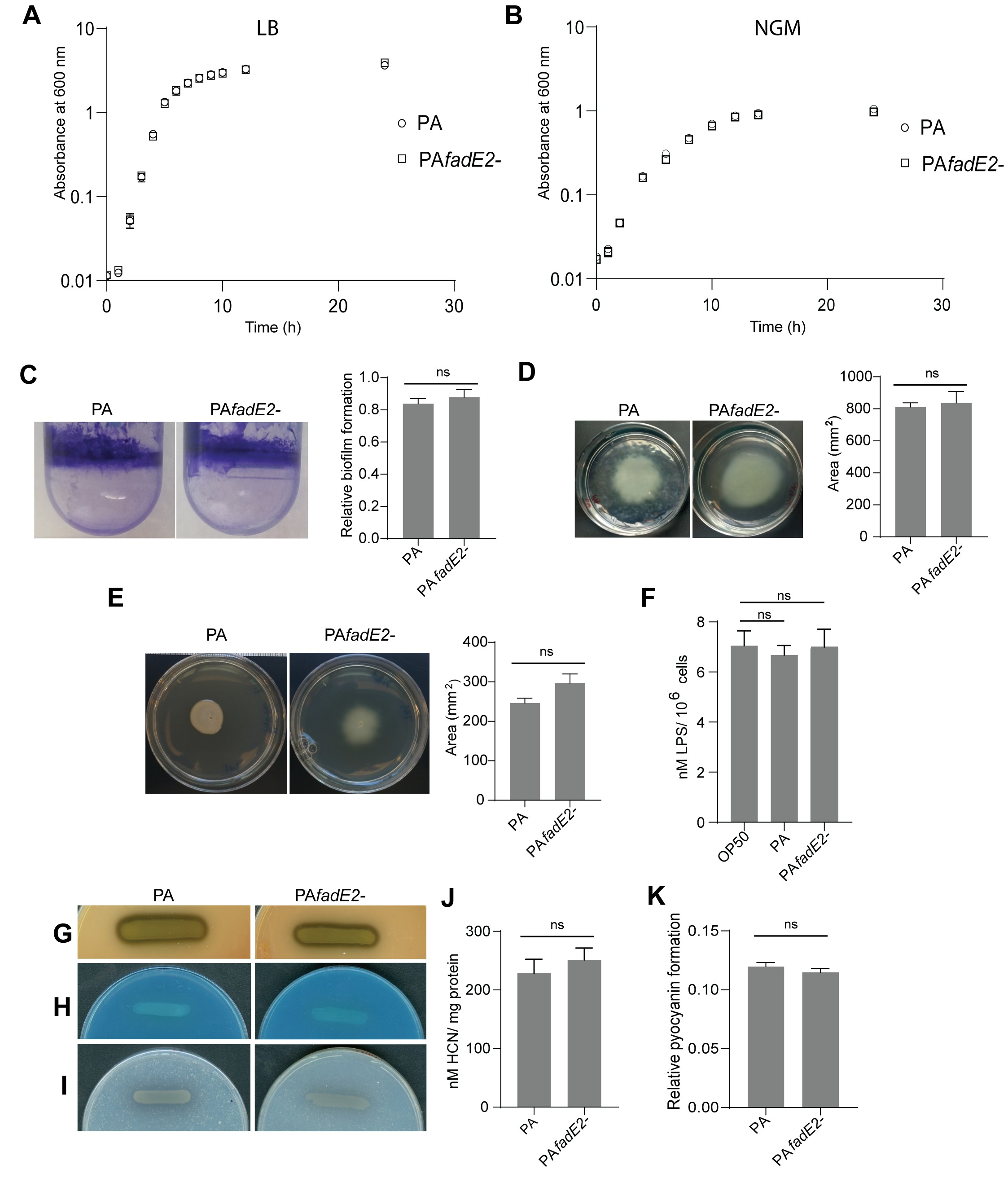

Supplement: S4 Fig — (A) Growth curves of wild-type P. aeruginosa (PA) or fadE2- in LB medium. (B) Growth curves of wild-type P. aeruginosa (PA) or fadE2- in NGM. (C) Photomicrographs and quantification of biofilm formation in wild-type P. aeruginosa (PA) or fadE2-. Shown is the mean ± SEM (n = 3). (D) Photomicrographs and quantification of swarming motility in wild-type P. aeruginosa (PA) or fadE2-. Shown is the mean ± SEM (n = 3). (E) Photomicrographs and quantification of twitching motility in wild-type P. aeruginosa (PA) or fadE2-. Shown is the mean ± SEM (n = 3). (F) Quantification of LPS levels in wild-type P. aeruginosa (PA) or fadE2-. Shown is the mean ± SEM (n = 3). (G-I) Photomicrographs showing production of (G) proteases, (H) rhamnolipids, and (I) elastase in wild-type P. aeruginosa (PA) or fadE2-. (J) Quantification of cyanide levels in wild-type P. aeruginosa (PA) or fadE2-. Shown is the mean ± SEM (n = 3). (K) Quantification of pyocyanin levels in wild-type P. aeruginosa (PA) or fadE2-. Shown is the mean ± SEM (n = 3). (C, D, E, F, J, K) ns denotes no significance using Student’s t-test. (TIF) [file ppat.1008918.s004.tif]

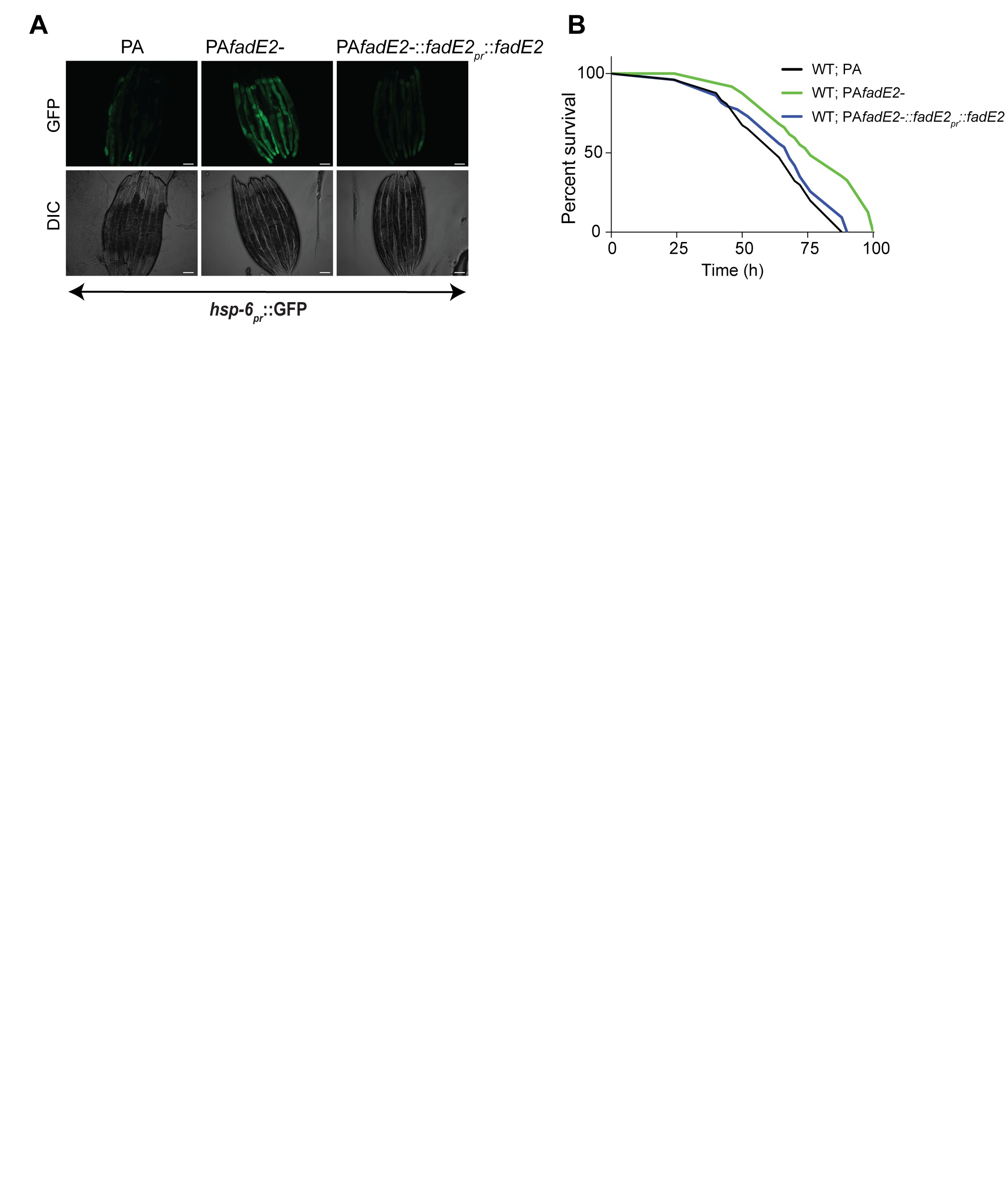

Supplement: S5 Fig — (A) hsp-6pr::GFP animals grown in the presence of wild-type P. aeruginosa (PA), fadE2-, or fadE2- expressing a FadE2 rescue plasmid for 48 hrs. (B) Survival of wild-type animals during infection with wild-type P. aeruginosa (PA), fadE2-, or fadE2- expressing the FadE2 rescue plasmid. See S2 Table for survival assay statistics. (TIF) [file ppat.1008918.s005.tif]

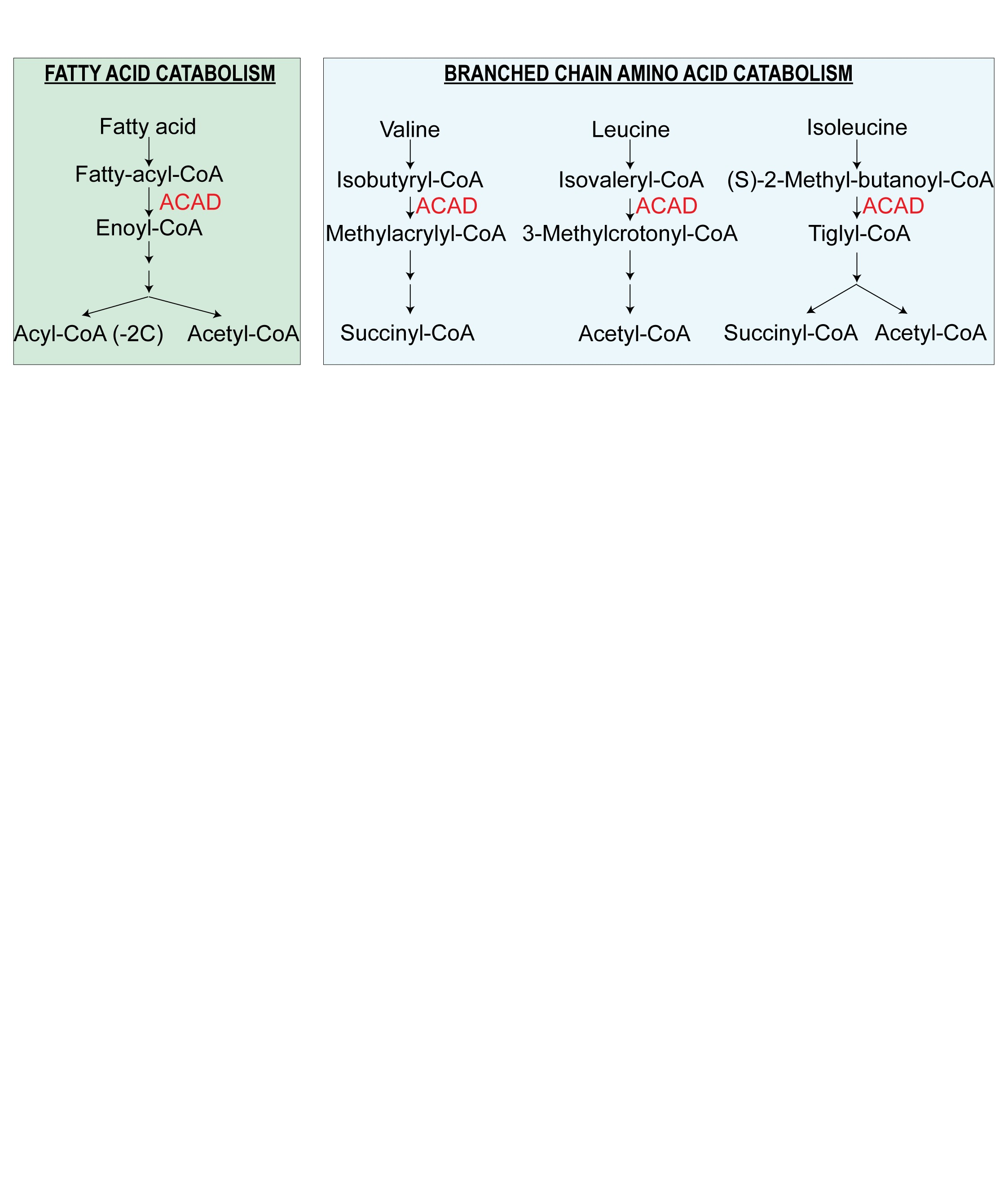

Supplement: S6 Fig — Schematic overview of the role of acyl-CoA dehydrogenases (ACAD) in fatty acid and BCAA catabolism. (TIF) [file ppat.1008918.s006.tif]

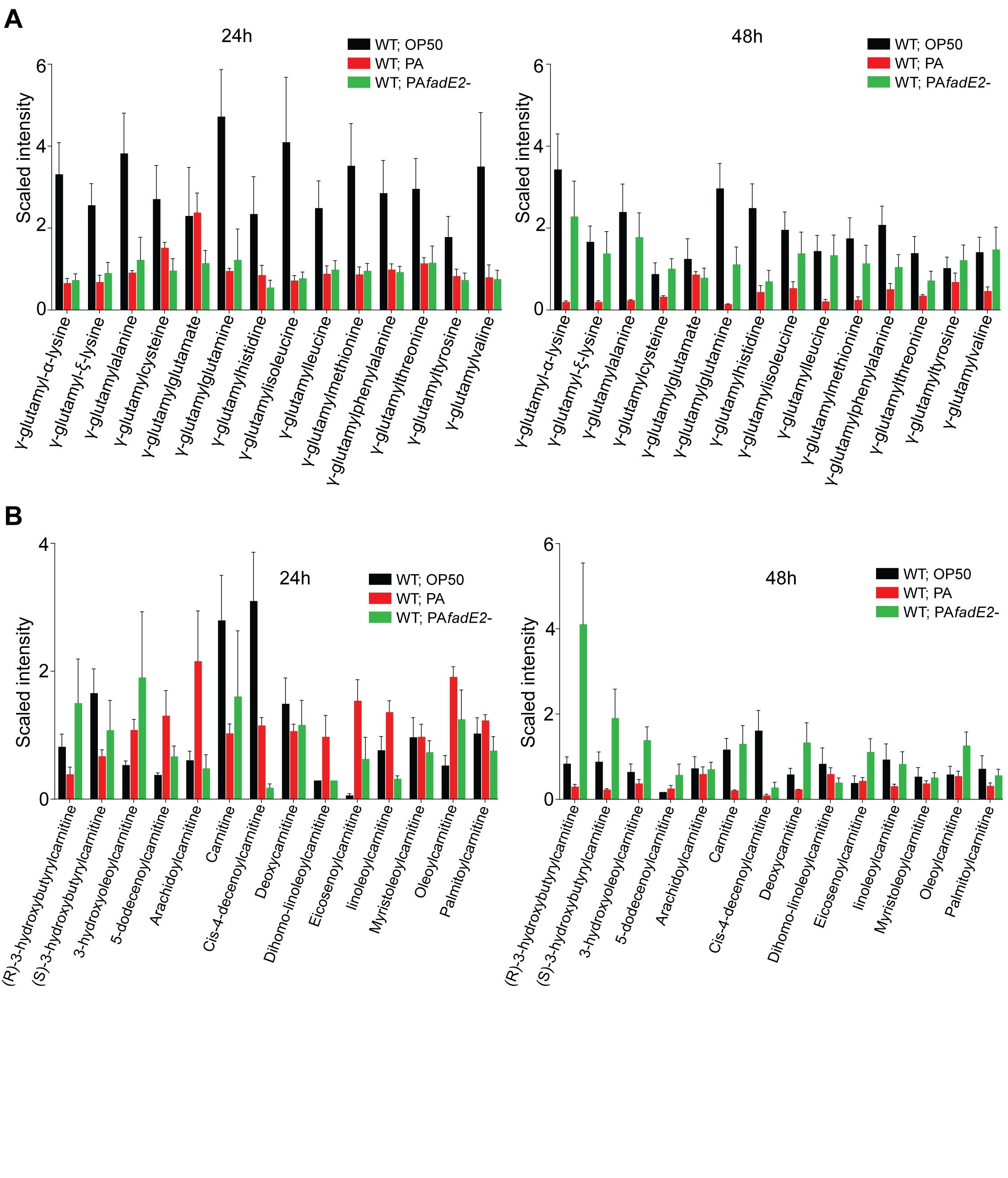

Supplement: S7 Fig — Quantification of metabolites related to (A) gamma-glutamyl amino acids, (B) carnitine metabolism by mass spectrometry using extracts of wild-type animals infected with wild-type P. aeruginosa (PA) or fadE2- following 24 or 48 hrs. Shown is the mean ± SEM (n≥4). (TIF) [file ppat.1008918.s007.tif]

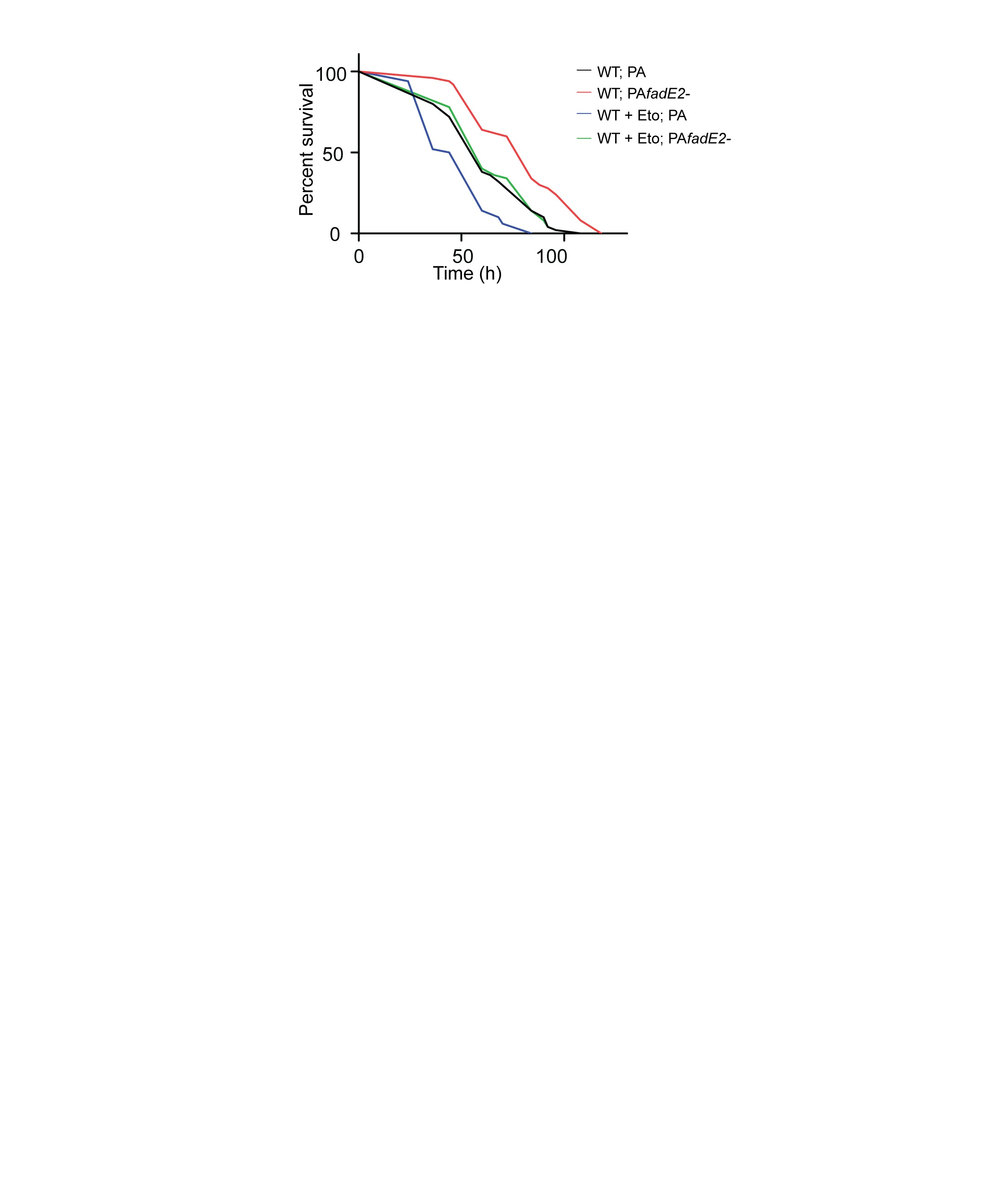

Supplement: S8 Fig — Survival of wild-type animals treated with 50 μM etomoxir (Eto) and infected with wild-type P. aeruginosa (PA) or fadE2-. See S2 Table for statistics. (TIF) [file ppat.1008918.s008.tif]

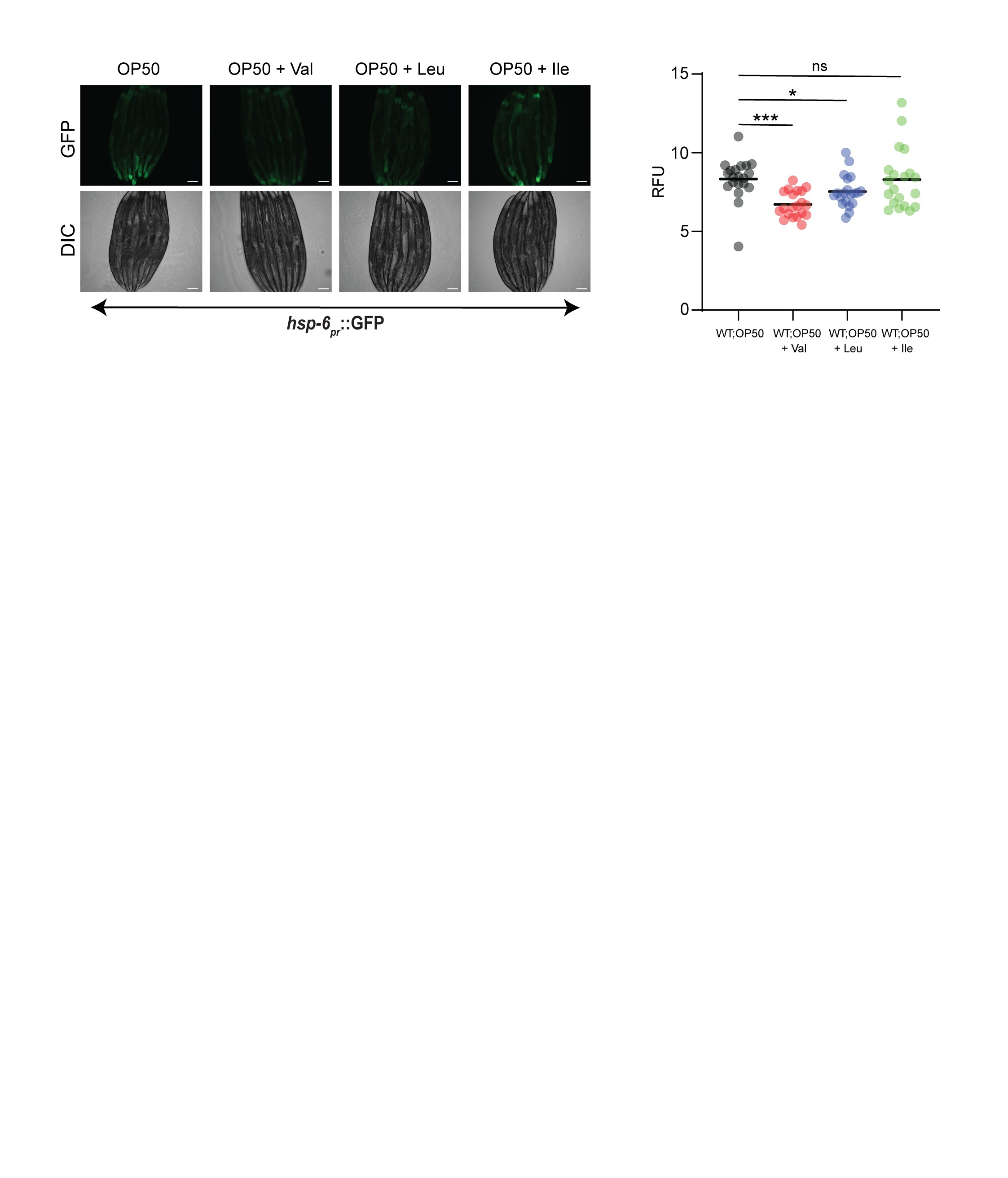

Supplement: S9 Fig — Photomicrographs and quantifications of hsp-6pr::GFP fluorescence for wild-type animals fed E. coli OP50 and supplemented with valine, leucine, or isoleucine. RFU: Relative Fluorescence Units. Shown is the mean ± SEM (n≥20 worms). Scale bar is 100 μm for all images. ns denotes no significance, *** denotes p<0.001, *denotes p<0.05 using Student’s t-test. (TIF) [file ppat.1008918.s009.tif]

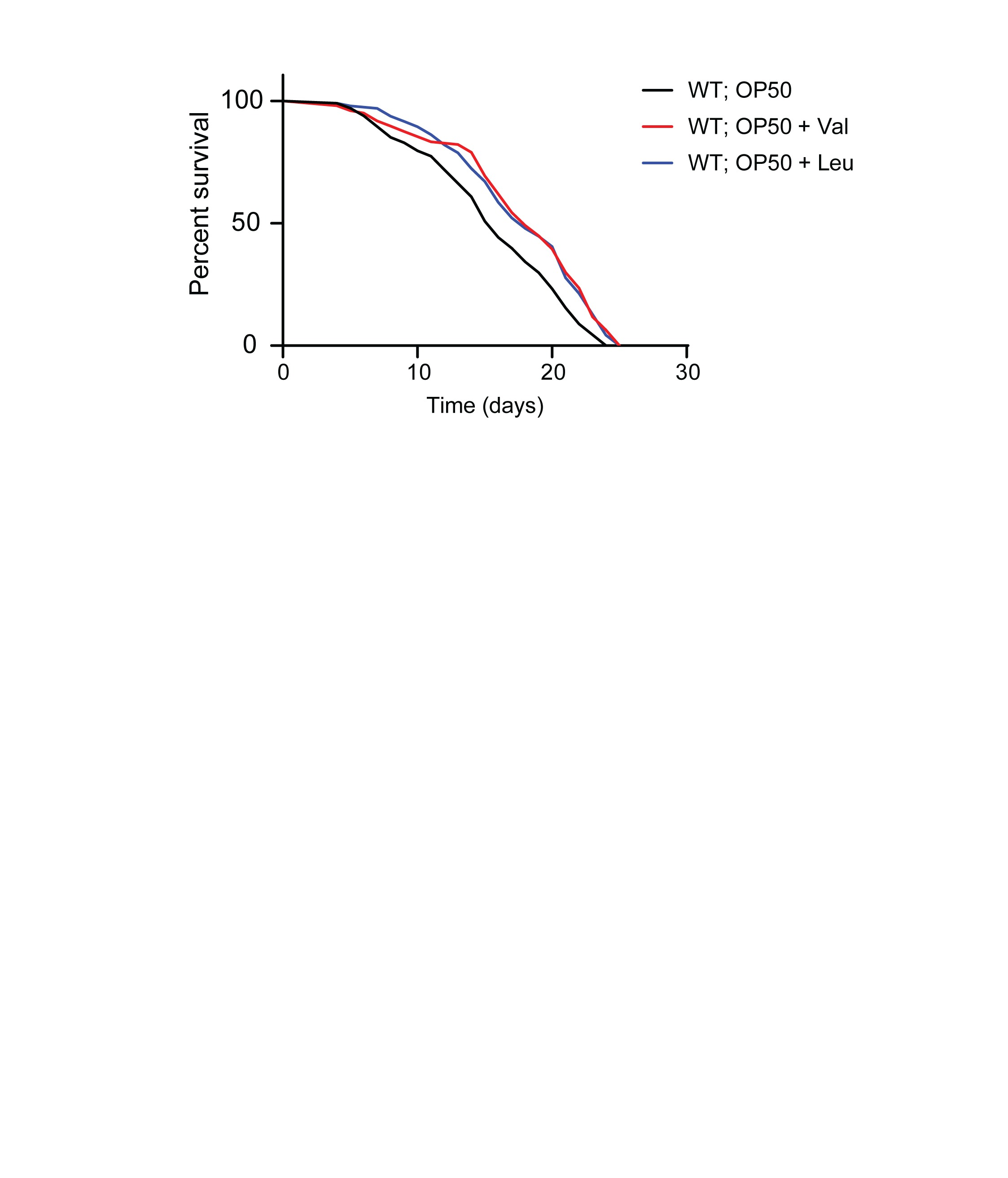

Supplement: S10 Fig — (A, B) Lifespans of wild-type animals supplemented with 5 mM (A) valine or (B) leucine fed a diet of E. coli OP50. (TIF) [file ppat.1008918.s010.tif]

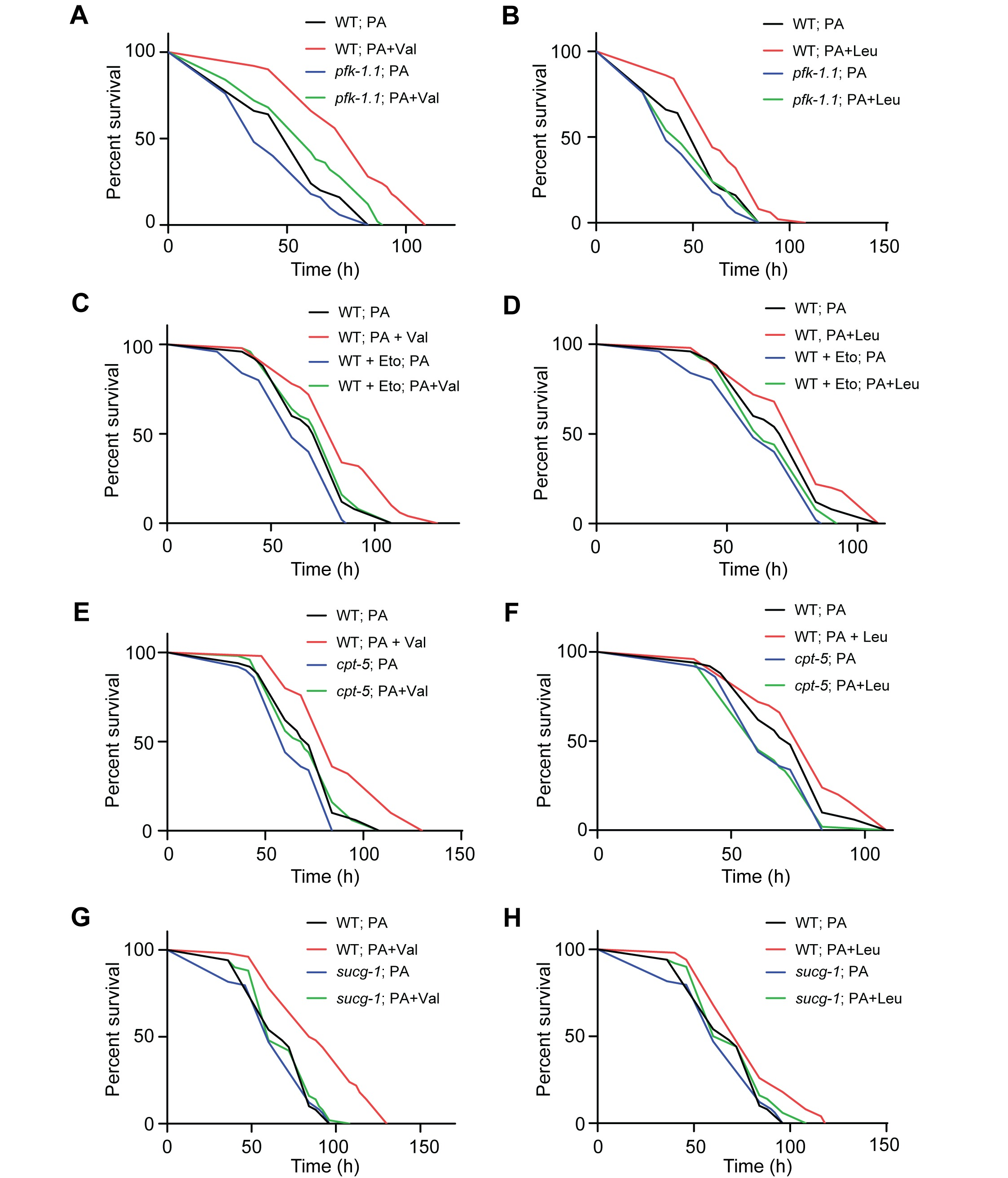

Supplement: S11 Fig — (A, B) Survival of pfk-1.1(ola72) animals infected with wild-type P. aeruginosa (PA) and supplemented with 5 mM (A) valine or (B) leucine. (C, D) Survival of wild-type animals treated with 50 μM etomoxir (Eto) and supplemented with 5 mM (C) valine or (D) leucine and infected with wild-type P. aeruginosa (PA). (E, F) Survival of cpt-5(gk5128) animals infected with wild-type P. aeruginosa (PA) and supplemented with 5 mM (E) valine or (F) leucine. (G, H) Survival of sucg-1(osa2) animals infected with wild-type P. aeruginosa (PA) and supplemented with 5 mM (G) valine or (H) leucine. (TIF) [file ppat.1008918.s011.tif]

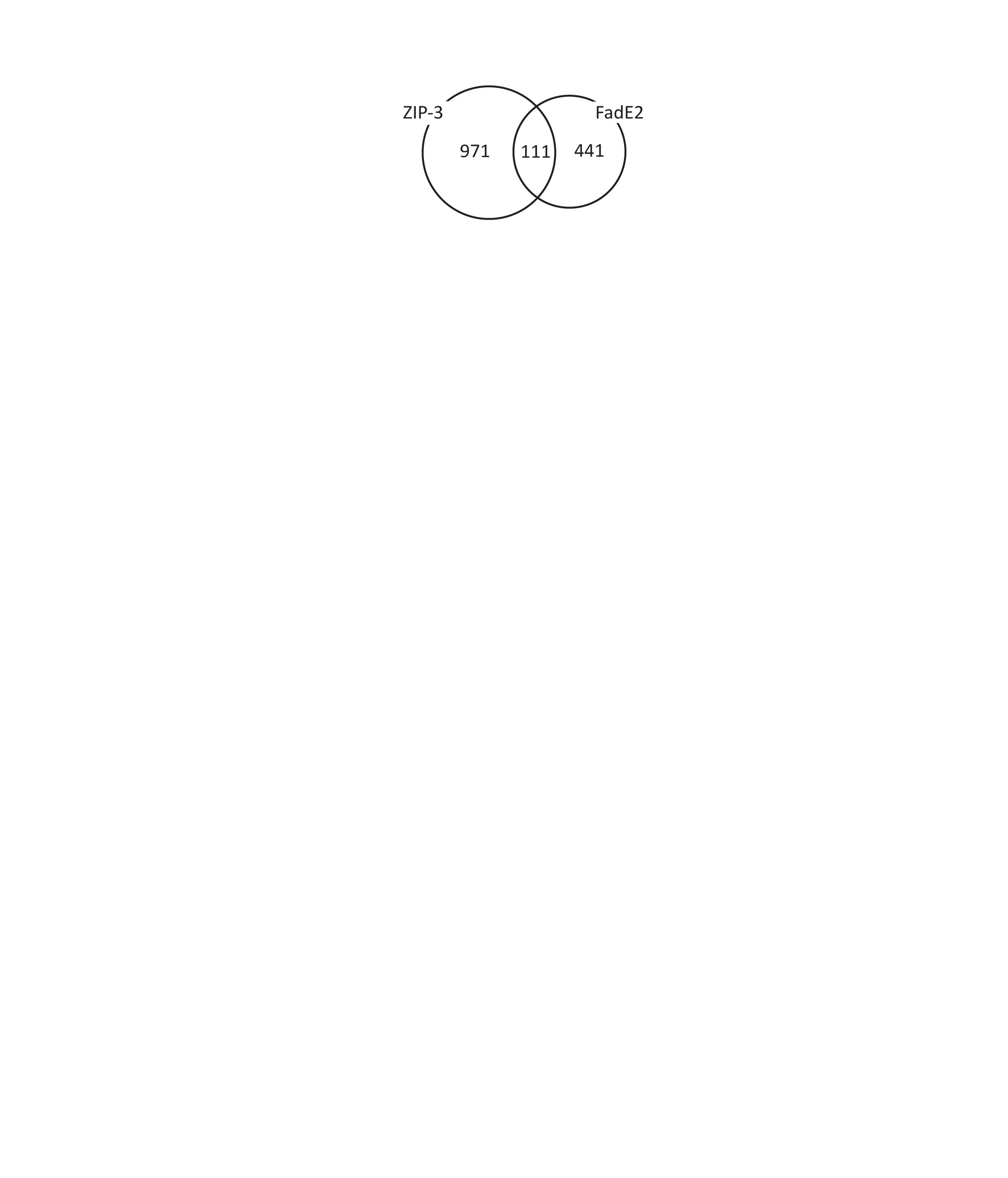

Supplement: S12 Fig — Venn diagram demonstrating the number of commons genes that are negatively regulated by P. aeruginosa FadE2 (see S3 Table) and ZIP-3 (see reference [15]). (TIF) [file ppat.1008918.s012.tif]
